# Prenatal exposure to per- and polyfluoroalkyl substances: Association with child behavior in the environmental influences on child health outcomes (ECHO) Cohort

**DOI:** 10.1016/j.envint.2025.109760

**Published:** 2025-08-30

**Authors:** Catherine M. Bulka, Lesliam Quiros-Alcala, Xiaoshuang Xun, T.Michael O’Shea, Joseph M. Braun, Jennifer L. Ames, Alison E. Hipwell, Vaia Lida Chatzi, Amy M. Padula, Dana Dabelea, Anne Starling, Anne L. Dunlop, Donghai Liang, Susan Schantz, Hyeong-Moo Shin, Jiwon Oh, Rebecca J. Schmidt, Kun Lu, Thomas G. O’Connor, Rebecca C. Fry

**Affiliations:** a College of Public Health, University of South Florida, Tampa, FL, USA; b Department of Environmental Health and Engineering, Bloomberg School of Public Health, Johns Hopkins University, Baltimore, MD, USA; c Environmental influences on Child Health Outcomes Data Analysis Center, Johns Hopkins University, Baltimore, MD, USA; d Department of Pediatrics, University of North Carolina School of Medicine, Chapel Hill, NC, USA; e Department of Epidemiology, Brown University, Providence, RI, USA; f Center for Children’s Environmental Health, Brown University, Providence, RI, USA; g Kaiser Permanente Division of Research, Oakland, CA, USA; h Department of Psychiatry, University of Pittsburgh, Pittsburgh, PA, USA; i Department of Population and Public Health Sciences, Keck School of Medicine, University of Southern California, Los Angeles, CA, USA; j Program on Reproductive Health and the Environment, Department of Obstetrics, Gynecology, and Reproductive Sciences, University of California, San Francisco, USA; k Lifecourse Epidemiology of Adiposity and Diabetes (LEAD) Center, University of Colorado Anschutz Medical Campus, Aurora, CO, USA; l Department of Epidemiology, Colorado School of Public Health, University of Colorado Anschutz Medical Campus, Aurora, CO, USA; m Department of Epidemiology, Gillings School of Global Public Health, University of North Carolina at Chapel Hill, Chapel Hill, NC, USA; n Department of Gynecology & Obstetrics, School of Medicine, Emory University, Atlanta, GA, USA; o Department of Environmental Health, Rollins School of Public Health, Emory University Atlanta, GA, USA; p Department of Epidemiology, Rollins School of Public Health, Emory University Atlanta, GA, USA; q Department of Psychology, College of Liberal Arts & Sciences, University of Illinois Urbana-Champaign, Champaign, IL, USA; r Department of Environmental Science, College of Arts & Sciences, Baylor University, Waco, TX, USA; s Department of Public Health Sciences, University of California Davis, Davis, CA, USA; t Department of Environmental Sciences and Engineering, Gillings School of Global Public Health, The University of North Carolina, Chapel Hill, NC, USA; u Department of Psychiatry, University of Rochester Medical Center, Rochester, NY, USA; v Institute for Environmental Health Solutions, Gillings School of Global Public Health, The University of North Carolina, Chapel Hill, NC, USA

**Keywords:** PFAS, Child development, Behavior, Pregnancy

## Abstract

**Background:**

Prenatal exposure to per- and polyfluoroalkyl substances (PFAS) may adversely impact child neurodevelopment; however, epidemiologic findings remain inconclusive because of small sample sizes, limited exposure variability, and differing neurodevelopmental measures.

We aimed to investigate the relationship between prenatal PFAS exposure and child behavior.

**Methods:**

We pooled data from nine study sites in the nationwide Environmental influences on Child Health Outcomes (ECHO) Cohort. PFAS were quantified in maternal serum samples collected between 2 and 42 weeks’ gestation. Behavioral and emotional problems were assessed via the Child Behavior Checklist (CBCL) in preschool-age (n = 1,723) and school-age (n = 627) children. We used age-stratified, covariate-adjusted linear mixed-effects models to estimate differences in CBCL scores by PFAS quartile, focusing on analytes detected at >75 %. We also fit quantile g-computation models to examine associations for PFAS mixtures and tested for effect modification by child sex.

**Findings:**

Perfluorooctane sulfonate (PFOS), perfluorooctanoic acid (PFOA), perfluorohexane sulfonate (PFHxS), and perfluorononanoic acid (PFNA) were the most frequently detected analytes in maternal prenatal serum, although concentrations were generally low (<5 ng/mL). Associations between PFAS concentrations and CBCL scores were mostly null, except for some suggestive findings for PFHxS in the preschool-age subset. No consistent sex differences were observed, and associations for PFAS mixtures were statistically insignificant.

**Interpretation:**

We found little evidence of associations between prenatal PFAS exposures and child behavioral problems in the ECHO Cohort. Future studies should consider PFAS exposure during the postnatal period, which may be a more sensitive window.

## 1. Introduction

Experimental studies in murine models have demonstrated that prenatal exposure to perfluoroalkyl and polyfluoroalkyl substances (PFAS) interfere with fetal brain development (Butenhoff et al., Jun 2009). These findings have raised concerns that exposure to these ubiquitous environmental chemicals may contribute to child behavioral problems (Liew et al., Mar 2018). PFAS are a large class of synthetic compounds widely used to make everyday consumer products more resistant to oil and water and can be found in building materials, clothing, personal care products, cookware, cleaning products, firefighting foams, and fabrics. Exposure to PFAS in the general population is known to occur mainly through consumption of contaminated food and drinking water (Profile, 2021). The developing fetus is exposed to PFAS via transplacental transfer, and infants can be further exposed through breastfeeding, ingestion of formula made with contaminated drinking water, and use of personal care and consumer products (Zheng et al., 2022; Glüge et al., 2020).

The exact mechanisms by which PFAS impact child neurodevelopment have not been clearly elucidated; however, preclinical studies have reported alterations in both maternal and offspring behavior linked to PFAS exposures. For example, in mouse studies, exposure to low doses of perfluorooctanoic acid (PFOA) during pregnancy results in “anxiety-like” behavior after delivery and atypical nursing postures that may have implications for mother-litter bonding (Merrill et al., 2022). Zebrafish larvae exposed to PFOA have shown disruption in dopamine synthesis and reuptake, which may be mediated by altered dopaminergic neuron development (Jantzen et al., Jun 2016). Furthermore, PFOS exposure in animals has been linked to altered hormone levels, including thyroid disruption, which can alter metabolism, growth, and central nervous system function (Fuentes et al., 2007). Prenatal and postnatal exposure to PFOS and PFOA has been linked to increased motor activity, decreased habituation, and deficits in spatial learning and memory abilities (Butenhoff et al., Jun 2009; Fuentes et al., 2007; Johansson et al., Jan 2008; Onishchenko et al., Apr 2011; Tukker et al., 2020).

Despite evidence from preclinical studies, epidemiologic findings on the role of PFAS in child neurodevelopment remain inconclusive (Tukker et al., 2020; Gao et al., 2023). Inconsistencies across epidemiologic studies may stem from small sample sizes, heterogeneity by child sex (Gao et al., 2023), biologic matrices used to assess PFAS exposure (Tillaut et al., Nov 2023), variability in the type and timing of neurodevelopmental measures, and limited geographic coverage of study populations as exposure profiles are highly location-dependent (DeLuca et al., Sep 2023). In the HOME study (N = 241) of mother-infant dyads from the greater Cincinnati area (United States [U.S.]), higher levels of perfluorooctane sulfonate (PFOS) and perfluorononanoic acid (PFNA), as measured in maternal serum during pregnancy or at delivery, were positively associated with child hyperactivity based on the Behavioral Assessment System for Children-2 (BASC-2) (Vuong et al., Apr 2021). A similar finding was observed in another U.S. cohort enriched for autism spectrum disorders, in which higher prenatal PFNA exposure was associated with more externalizing problems and aggressive behaviors at age 3 according to the Child Behavior Checklist (CBCL) (Choi et al., 2024). In contrast, a cohort study composed of mother-infant dyads from eastern Massachusetts (N = 1,080) reported that prenatal exposure to PFOS, PFNA, and PFHxS, as measured in maternal plasma during pregnancy, was unrelated to behavioral problems in mid-childhood based on the Strengths and Difficulties Questionnaire (SDQ) (Harris et al., Nov 2021). Other studies have focused on simultaneous prenatal exposures to multiple PFAS in relation to child behavioral problems and reported increased externalizing problems in early childhood (Xie et al., Aug 2022; Xie et al., 2023).

The inconclusive associations between prenatal PFAS exposures and child behavior warrant further investigation in larger populations. In this study, we examined the relationship between prenatal PFAS exposure and children’s behavior by leveraging data collected from multiple sites across the U.S. in the National Institutes of Health-funded Environmental influences on Child Health Outcomes (ECHO) Cohort. As such, this study is one of the largest and most geographically diverse U. S.-based studies of early-life PFAS exposure and behavior to date, with behavioral problems assessed during two distinct developmental stages—early childhood and middle/late childhood through adolescence—using the CBCL, a highly reliable, valid, and culturally appropriate tool (Ivanova et al., 2007; Achenbach et al., Mar 1987; Achenbach and Edelbrock, 1981).

## 2. Methods

### 2.1. Study population

We evaluated prenatal PFAS concentrations in serum and CBCL data from nine ECHO Cohort study sites (Knapp et al., 2023), including Chemicals in Our Bodies (CiOB, California; 2014–2019), Illinois Kids Development Study (IKIDS, Illinois; 2014–2020), Magee Cohort (Pennsylvania; 2017–2020), Maternal and Development Risks from Environmental and Social Stressors (MADRES, California; 2016–2019), Atlanta ECHO Cohort of Emory University (Georgia; 2014–2018), Conditions Affecting Neurocognitive and Learning in Early Childhood (CANDLE, Tennessee; 2007–2011), Healthy Start (Colorado; 2010–2014), Archive for Research in Child Health (ARCH, Michigan; 2009–2017), and Pregnancy Environment and Lifestyle Study (PETALS, California; 2014–2017). The samples were geographically diverse, with participants drawn from seven states across various regions of the U.S., including the Mid-Atlantic, Midwest, South Atlantic, Mountain and Pacific West. The preschool-age children ranged from 1.9–5.9 years of age, whereas the school-age children ranged from 6–15 years of age. All study sites received institutional review board approval from their local institutions. Additionally, all sites obtained written informed consent from all parents and observed the privacy rights of all participants. Of note, while most sites restricted their inclusion criteria to singleton pregnancies and allowed only one child from each family to enroll, some did not. In these instances, we randomly selected one child participant to ensure that each mother was only represented once in the dataset.

### 2.2. PFAS exposure assessment

While all sites collected maternal serum during pregnancy, the gestational age at sample collection differed. Approximately 19 % of participants had maternal serum during the first trimester (≤13 weeks), 56 % during the second trimester (>13 and ≤ 26 weeks), and 25 % during the third trimester (>26 weeks). Across all study sites, prenatal PFAS serum samples were frozen after collection, then shipped with dry ice to one of three laboratories for PFAS quantification: the California Department of Toxic Substances Control (California, Eick et al., 2021) Centers for Disease Control and Prevention (CDC; Georgia, Kato et al., 2011) or Human Health Exposure Analysis Resource (HHEAR, New York, Honda et al., 2018). Across all three laboratories, PFAS were quantified in nanograms per milliliter (ng/mL) using liquid chromatography with mass spectrometry. All laboratories participated in the CDC quality assurance program to test interlaboratory comparisons, in addition to testing quality control materials, analytical standards, and reagent and serum blanks along with participant samples to ensure accuracy and reliability (Kato et al., 2011; Honda et al., 2018; Starling et al., 2017; Morello-Frosch et al., 2016). The number of PFAS compounds measured differed by the study site, ranging from 10 to 15. If a site measured branched- and linear-chain isomers for PFOA or PFOS separately, the two values were summed to calculate the total PFOA or PFOS concentration. More detailed information on maternal PFAS measurements by individual ECHO Cohort study sites is provided in Table S1.

Some study sites collected repeated serum samples in different trimesters for a limited number of participants (first and second, N = 69; second and third, N = 291; first and third, N = 5), although no participants had samples collected across all three trimesters. For participants with repeated PFAS measurements across any two trimesters, we calculated intraclass correlation coefficients (ICCs) and found relatively high ICC values for most compounds (i.e., ICCs > 0.8, Table S2). Therefore, for these participants, mean serum concentrations were calculated and used as their individual PFAS exposure measure. Of note, there were two PFAS (PFNA and PFOA) with ICCs < 0.8 that were addressed in sensitivity analyses, as described in the ‘Statistical Analyses’ section. For all maternal serum PFAS measurements, non-detectable concentrations were substituted with the limit of detection divided by the square root of two (Hornung and Reed, 1990).

### 2.3. CBCL Outcome assessment

Parent/child report on the CBCL was used to measure child behavioral problems. The CBCL is a reliable and valid instrument across diverse cultures (Ivanova et al., 2007; Achenbach et al., Mar 1987; Achenbach and Edelbrock, 1981), and as such, it is the most widely used tool for the early detection of behavioral problems in children (Cianchetti, 2020). We obtained scores from two versions of the CBCL based on the age of the study participant: the preschool CBCL (CBCL/1½−5) for children 1.9–5.9 years of age and the school-age CBCL (CBCL/6–18) for children 6–15 years of age. Each version provides quantitative measures of behavioral problems across different domains. The preschool version consists of 99 items while the school-age version contains 113 items that describe the child’s current or recent behaviors. Respondents are instructed to rate each statement on a Likert scale from “not true” (0), “somewhat true” (1), or “very true” (2).

It should be noted that the CBCL is a general screening tool meant to detect behavioral and emotional problems in children. It is not a diagnostic tool, although scores have been reported to predict clinical diagnosis of conditions, such as attention-deficit/hyperactivity disorder (ADHD) and anxiety/depression (Aebi et al., Mar 2010; Biederman et al., Oct 2005). The preschool version of the CBCL captures 7 distinct syndromes (emotional reactivity, anxiety/depression, somatic complaints, withdrawn, sleep problems, attention problems, and aggressive behavior), whereas the school-age version captures 8 syndromes (anxiety/depression, withdrawn, somatic complaints, social problems, thought problems, attention problems, rule-breaking behavior, and aggressive behavior), with higher scores indicative of more behavioral problems. The syndrome scores were combined to quantify internalizing problems (behaviors directed inwardly toward oneself), externalizing problems (behaviors directed toward the external environment), and total problems, which served as our primary outcomes of interest, with the individual syndrome scores as secondary.

We calculated normalized T-scores according to the child’s sex and age (mean: 50; standard deviation: 10) using normative data obtained from the CBCL developer. Both the normalized CBCL T-scores and raw CBCL scores were analyzed as continuous endpoints. T-scores, which compress raw scores within the normal range, may allow for the detection of more clinically relevant relationships between prenatal PFAS exposures and child behavior, especially in study populations that include children of varied ages, such as in this study, whereas raw scores may allow for the detection of more subtle associations (Drotar et al., Jun 1995). For the present analyses, prenatal maternal PFAS concentrations were available for two mutually exclusive groups: the 1,723 children who were administered the preschool CBCL and for the 627 children who were administered the school-age CBCL.

### 2.4. Covariates

Potential confounders were identified and selected based on the prior epidemiologic literature and a directed acyclic graph (DAG; Fig. S1). The minimally sufficient adjustment set included maternal age (continuous, years), race and ethnicity (non-Hispanic White, non-Hispanic Black, non-Hispanic Asian, non-Hispanic Other Race, Hispanic), educational attainment (less than high school, high school diploma, General Educational Development (GED) or equivalent, at least some college or more), marital status (married or living with a partner, single/separated), pre-pregnancy body mass index (BMI) (continuous, kg/m^2^), parity (nulliparous, parous), and child birth year (continuous, range: 2007–2020). Maternal educational attainment was used as a proxy for socioeconomic status due to the high level of missingness for household income. Maternal smoking during pregnancy (yes, no) was considered a potential confounder but was omitted from the primary models due to high missingness of data. Instead, this potential confounder was considered in separate models as part of our sensitivity analyses. Additionally, all models included child sex (male, female) and age at CBCL assessment (continuous, years) as precision variables; random intercepts were further incorporated to account for clustering by ECHO study site. Data on most covariates, including age, sex, race and ethnicity, educational attainment, marital status, smoking status, and parity, were self-reported, whereas maternal BMI was abstracted from medical records when available.

### 2.5. Statistical analyses

All statistical analyses were stratified by the CBCL version that corresponded to the child’s age group at the time of the assessment: preschool (1.9–5.9 years) or school-age (6–15 years). Each version of the CBCL ascertained distinct problem behaviors. We first estimated summary statistics for participant characteristics and PFAS concentrations. We used Wilcoxon rank-sum tests to examine differences by sex for PFAS concentrations and CBCL scores, which both had skewed distributions. To examine associations for individual PFAS measurements, we fit linear mixed-effects models for both the normalized CBCL T-scores and raw CBCL scores. For all models, PFAS concentrations were categorized into four levels, and we additionally calculated *p*-values for linear trends by treating the levels as ordinal variables. For PFAS detected in more than 75 % of the samples, the four levels corresponded to quartiles; these PFAS were the primary exposures of interest for this study. For PFAS detected in 50 %−75 % of the samples, the lowest level included all samples below the limit of detection (LOD), with the remainder of detectable concentrations divided into tertiles; these PFAS were of secondary importance due to their infrequent detection. However, in additional sensitivity analyses of both the primary and secondary PFAS analytes, we re-ran all models per a 1-ng/mL increase (continuous variables), rather than as categorical variables.

To examine associations for mixtures of PFAS, we fit quantile g-computation models to serum concentrations and normalized CBCL T-scores and raw scores for internal, external, and total behavioral problems among the preschool-age and school-age subsets (Keil et al., Apr 2020). Only PFAS analytes with a detection frequency >75 % were included in the mixtures analyses. These models were adjusted for the same covariates as the individual PFAS models (ECHO Cohort study site, maternal age, race and ethnicity, educational attainment, marital status, pre-pregnancy BMI, year of birth, child sex and age at the time of CBCL assessment, all as fixed effects).

Previous epidemiologic studies have reported that prenatal exposures to PFAS may exert sexually dimorphic effects on neurodevelopmental outcomes (Gao et al., 2023). To assess effect-measure modification by sex, we added a cross-product term between the categories of each PFAS compound and binary sex to the adjusted models that associated prenatal PFAS concentrations with aggregate CBCL scores (i.e., Internalizing Problems, Externalizing Problems, and Total Problems).

All statistical analyses were conducted in R (version 4.2.2). Overall, the statistical significance criterion was set at *p* < 0.05 for all main analyses and at *p* < 0.10 for effect-measure modification by sex due to the exploratory nature of sex-specific analyses and the reduced number of observations within each sex stratum. We did not correct for multiple comparisons based on the few *a priori* tests conducted and focused on presenting actual observations (Rothman, Jan 1990). We used a complete-case approach for all analyses, such that participants with missing data on relevant covariates were excluded, and we did not report cell sizes smaller than five to protect confidentiality. Additionally, we did not model associations with child behavior for any PFAS that had a detection rate <50 %.

In addition to these sensitivity analyses (i.e., the inclusion of maternal smoking during pregnancy as an additional model covariate and the modeling of maternal prenatal serum PFAS concentrations as continuous variables), we performed several other sensitivity analyses. Specifically, we conducted select leave-one-out analyses of models of highly detected, individual PFAS with normalized CBCL scores to examine whether individual study sites influenced any statistically significant results. In addition, for PFNA and PFOA, which had ICCs below 0.80 (indicating poor reliability throughout pregnancy), we re-ran individual PFAS-normalized CBCL T-score regression models using only measurements from samples collected during the second trimester. For results from quartile-based models that were suggestive of nonlinear associations, we fit additional models using cubic splines with 3 knots to calculate *p*-values for nonlinearity. Finally, for associations between highly detected PFAS modeled using quartiles and CBCL scores that appeared to be non-linear, we calculated *p*-values for non-linearity using cubic splines with 3 knots.

## 3. Results

### 3.1. Study participants

A flow diagram depicting the derivation of the final analytic samples is shown in Fig. S2. After excluding participants with missing data on relevant covariates, a total of 1,505 dyads (out of a possible 1,723) were included in analyses of the preschool version of the CBCL and 581 dyads (out of a possible 627) were included in analyses of the school-age version. Sample characteristics are reported in Table 1; sample characteristics of those excluded due to data missingness are reported in Table S3. In general, the excluded individuals were similar to those included in the final analytic samples. However, the children excluded from the preschool-age subset tended to be younger than those who were included. Additionally, the mothers of the children in the school-age subset who were excluded tended to be younger, represented racial and ethnic minority groups, and had a lower income.

Nearly all CBCLs were completed by the biological mother (n > 1,500 for the preschool-age subset; n > 575 for the school-age subset); however, a small number were completed by biological fathers (n < 5 for the preschool-age subset; n < 5 for the school-age subset) or the child themselves (n < 5 for the school-age subset).

In general, the preschool-age subset of children was similar to the school-age subset with respect to maternal sociodemographic characteristics. One notable difference among participants with income data was that the school-age group had lower household incomes, with 62 % reporting an annual income below $30,000 compared with only 40 % in the preschool-age group.

### 3.2. Serum PFAS concentrations

Of the PFAS biomarkers measured in prenatal maternal serum from the preschool-age subset, four were detected in more than 75 % of the participants, namely PFHxS (99 %), PFOS (98 %), PFNA (96 %), and PFOA (80 %; Table 2). Respective geometric means (geometric SDs) were: 1.03 (2.72) ng/mL for PFHxS, 2.47 (2.53) ng/mL for PFOS, 0.29 (2.66) ng/mL for PFNA, and 0.69 (2.86) ng/mL for PFOA. Four other PFAS were also detected in at least half of the participants, including perfluorodecanoic acid (PFDA) (57 %), N-methylperfluorooctane sulfonamidoacetic acid (NMFOSAA or MeFOSAA) (55 %), perfluoroundecanoic acid (PFUNDA) (53 %), and perfluoropentanoic acid (PFPEA) (52 %; Table S4). Distributions of other PFAS compounds detected less frequently (<50 % of samples) are provided in Table S4. We observed no significant differences in prenatal maternal serum PFAS concentrations by child sex among the preschool-age subset.

Within the school-age subset, the three most frequently detected PFAS biomarkers in prenatal maternal serum samples were PFHxS (98 %), PFOS (99 %), and PFNA (99 %) (Table 2). Respective geometric means (geometric SDs) were: 1.44 (2.63) ng/mL for PFHxS, 4.13 (2.24) ng/mL for PFOS, and 0.51 (1.88) ng/mL for PFNA. Other PFAS biomarkers detected in 50 %−66 % of the mothers included PFOA (66 %), PFDA (53 %), NMFOSAA or MeFOSAA (54 %), PFUNDA (50 %), and N-Ethylperfluorooctanesulfonamidoacetic acid (EtFOSAA) (54 %). Distributions for these and other less frequently detected PFAS are provided in Table S4. Similar to the preschool subset, no sex-based differences in serum PFAS concentrations were observed.

The prenatal maternal PFAS levels observed across the nine ECHO Cohort study sites were lower than those reported in other studies of childhood behavior in the U.S. (Table S5), which may be due to the increased geographic representation in the ECHO Cohort and the inclusion of more recent pregnancies (2007 through 2020), some of which occurred after the phase-out of U.S.-based production of PFHxS, PFOS, and PFOA.

### 3.3. CBCL scores

Median CBCL scores (both normalized and raw) are provided along with interquartile ranges (25th, 75th percentiles) in Table 3. Median T-scores for total behavioral problems were below the age- and sex-normalized values of 50. However, some sex differences were observed, with males tending to score higher than females, which indicates more behavioral problems.

### 3.4. Associations between serum PFAS and CBCL scores

**Fig. 1.**
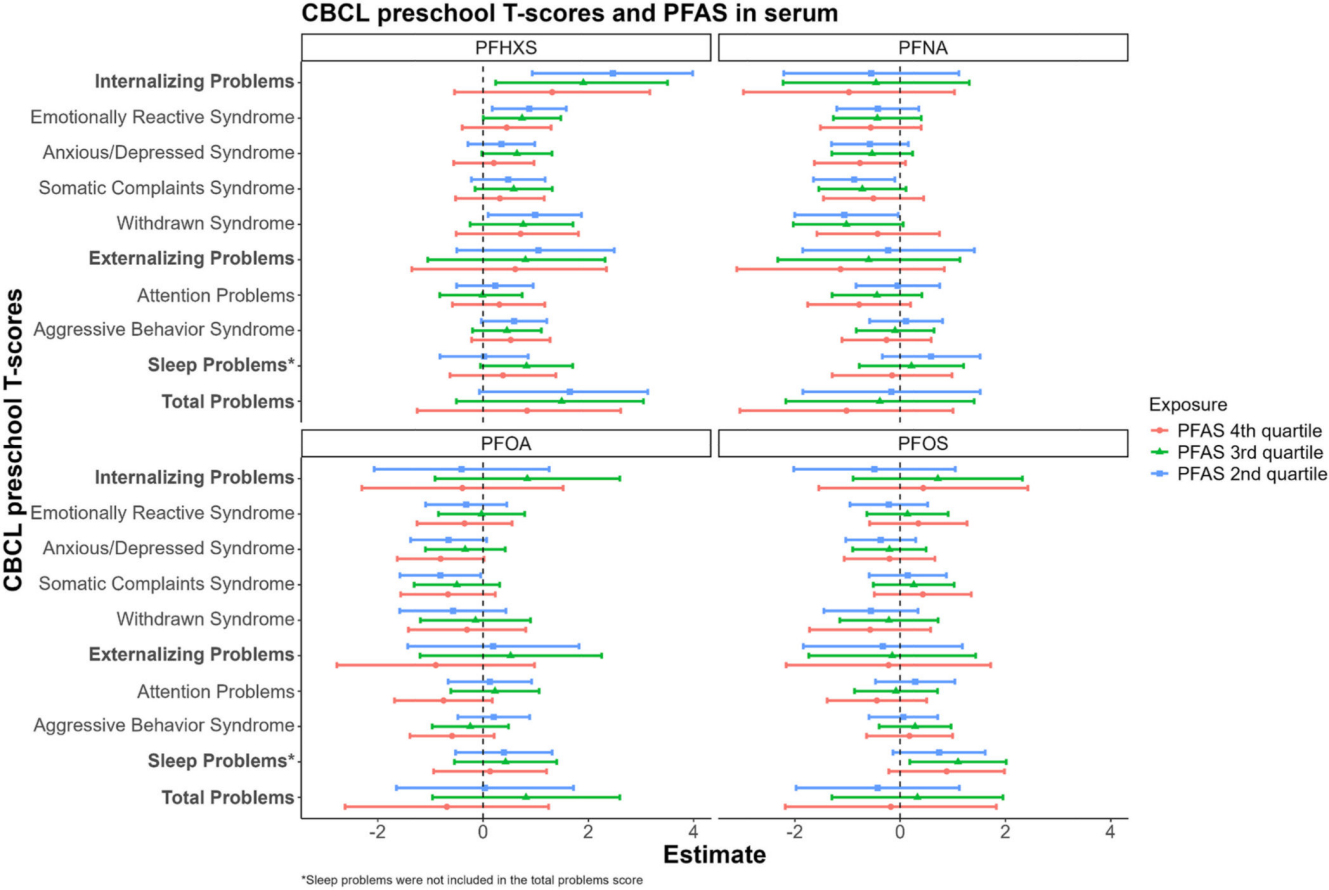
Adjusted associations between PFAS detected at > 75 % frequency in maternal prenatal serum and normalized CBCL T-scores among preschool-age children 1.9–5.9 years old. All models were adjusted for child sex, child age at CBCL assessment, birth year, maternal age at delivery, maternal race and ethnicity, maternal pre-pregnancy body mass index (BMI), maternal educational attainment, parity, marital status, and ECHO Cohort study site. CBCL, Child Behavior Checklist; ECHO, Environmental influences on Child Health Outcomes; PFAS, per- and polyfluoroalkyl substances.

Associations of both normalized and raw CBCL scores in the preschool-age subset were largely null for serum PFNA, PFOA, and PFOS when analyzed categorically, including analyses of PFNA and PFOA restricted to second-trimester samples (Fig. 1, Fig. S3, Fig. S4). Mid-range serum concentrations of PFHxS were associated with higher total behavioral problem scores (both normalized and raw) among the preschool-age children. For instance, concentrations in the 2nd quartile were associated with a 1.65 (95 % CI: − 0.07, 3.13) higher T-score and a 2.72 (95 % CI: 0.03, 5.41) higher raw score, whereas concentrations in the 3rd quartile were associated with a 1.49 (95% CI: − 0.51, 2.05) higher T-score and a 2.81 (95 % CI: − 0.04, 5.65) higher raw score relative to the lowest quartile. The point estimates for the 4th quartile were not statistically significant (β for total behavioral problem T-scores: 0.84, 95 % CI: − 1.25, 2.61; β for total behavioral problem raw scores: 1.17, 95 % CI: − 2.14, 4.47). These non-monotonic associations (p_trend_ = 0.47 for T-scores; p_trend_ = 0.29 for raw scores) were more frequently observed in associations with internalizing behavioral problems, as opposed to externalizing. With respect to the specific syndromes that compose internalizing behavioral problems, mid-range prenatal serum PFHxS concentrations were consistently associated with more emotional reactivity and withdrawn behavior across both the normalized and raw scores (Fig. 1, Fig. S3). P-values obtained from cubic spline models (Table S6) supported nonlinear associations of PFHxS with internalizing problems (*p*_nonlinearity_ < 0.01), emotional reactivity (*p*_nonlinearity_ = 0.08), withdrawn behavior (*p*_nonlinearity_ = 0.03), and total behavioral problems (*p*_nonlinearity_ = 0.04). When analyzed as continuous variables, associations were generally null except for a few inverse associations that were observed with raw scores, including PFOS with attention problems and PFNA with anxiety/depression and sleep problems (Table S6). For PFAS analytes with lower detection rates among the preschool-age children (i.e., PFDA, NMFOSAA/MeFOSAA, PFUNDA, and PFPEA), associations with CBCL scores were mostly null, except for NMFOSAA/MeFOSAA concentrations in the first tertile above the LOD, which were associated with lower CBCL scores (Figs. S5 and S6).

**Fig. 2.**
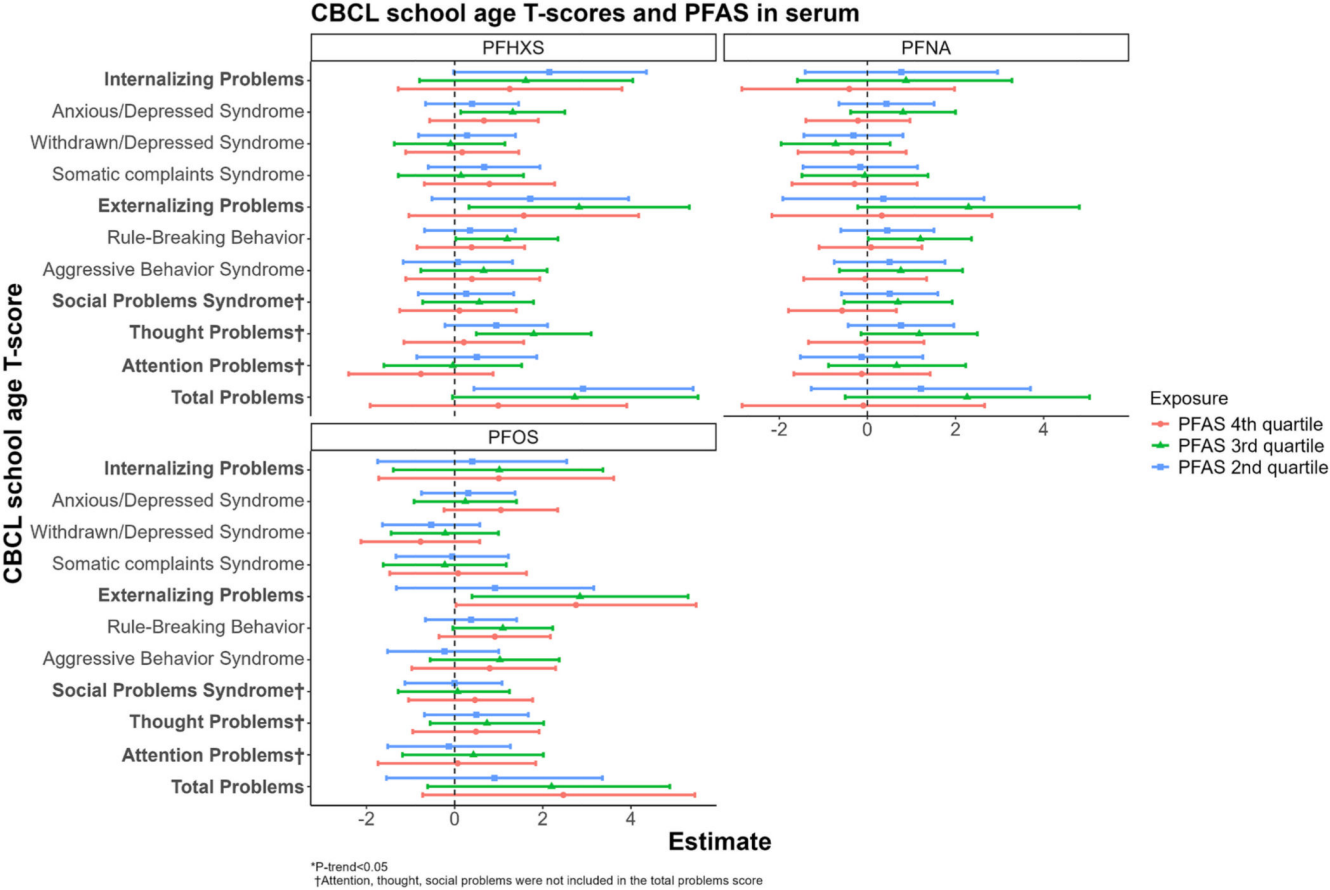
Adjusted associations between PFAS detected at > 75 % frequency in maternal prenatal serum and normalized CBCL T-scores among school-age children 6–15 years old. All models were adjusted for child sex, child age at CBCL assessment, birth year, maternal age at delivery, maternal race and ethnicity, maternal pre-pregnancy body mass index (BMI), maternal educational attainment, parity, marital status, and ECHO Cohort study site. CBCL, Child Behavior Checklist; ECHO, Environmental influences on Child Health Outcomes; PFAS, per- and polyfluoroalkyl substances.

For the school-age subset, we did not observe consistent associations between prenatal serum PFHxS, PFOS, and PFNA concentrations (including in models of second-trimester PFNA concentrations) with normalized and raw CBCL scores (Fig. 2, Fig. S7, Fig. S8). For example, PFHxS concentrations in the 2nd quartile were associated with higher T-scores for total behavioral problems and externalizing behavioral problems (Fig. 2); however, they were not significantly associated with the corresponding raw scores (Fig. S7), and models using cubic splines did not show significant improvements in model fit (Table S6). Additionally, PFOS concentrations in the 3rd and 4th quartiles were significantly associated with higher T-scores for externalizing behavioral problems (Fig. 2, β for Q3: 2.84, 95 % CI: 0.39, 5.30; β for Q4: 2.75, 95 % CI: 0.03, 5.48), but were not significantly associated with higher raw scores (Fig. S7). When analyzed per a 1 ng/mL-increase, associations were near null with wide confidence intervals, except for PFNA, which was associated with higher withdrawn behavioral scores (Table S7).

For PFDA, NMFOSAA/MeFOSAA, and PFUNDA, which were detected in 50 %−55 % of samples from the school-age subset, we again observed mostly null associations with CBCL scores (Figs. S9 and S10). For PFOA, which was detected in 66 % of samples from the school-age subset (Table 2), higher CBCL scores for aggregate behavioral problems and some syndromes (e.g., aggressive behavior) were observed for children with maternal serum concentrations in the second and third quartiles relative to the lowest; however, the confidence intervals were markedly wider for T-scores compared with raw scores (Figs. S9 and S10). The results from models using only second-trimester PFOA concentrations showed similar elevations of T-scores when comparing the second and third quartiles with the lowest quartile (Fig. S8).

Among the subsets with data available on maternal smoking during pregnancy, the inclusion of this covariate in the model did not meaningfully change the results (Table S7). In models with interaction terms for PFAS and child sex, we found no evidence of heterogeneity (all p_sex interaction_ > 0.10, Table S8). Implementing leave-one-out analyses did not alter the inference of our results nor indicate that any given study site was highly influential (Figs. S11–S13). Lastly, the quantile g-computation results for both the preschool- and school-age subsets showed no associations between mixtures of frequently detected PFAS and normalized or raw CBCL scores (Table 4).

## 4. Discussion

In this study, we leveraged data from children participating in nine ECHO Cohort study sites across the U.S., making it among the largest, most geographically-diverse study of early-life PFAS exposures and child behavior to date. We found that most of the PFAS highly detected in prenatal maternal serum were inconsistently associated with child behavioral problems as measured by the CBCL. Additionally, the overall associations for mixtures of legacy PFAS compounds were null and no effect modification by sex was observed. However, there were some suggestive results for PFHxS in the preschool-age subset, such that mid-range maternal prenatal serum concentrations were associated with more internalizing behavioral problems.

The levels of PFAS detected in prenatal maternal serum were generally lower than those observed in other recent epidemiologic studies examining associations with child behavior outcomes. The exposure levels in the current study were similar to those in the MARBLES cohort (N = 280) from California and the Maternal-Infant Research on Environmental Chemicals (MIREC) study (N = 757) based in Canada (Choi et al., 2024; Saha et al., Jan 2025). In the former, greater prenatal exposure to PFNA was associated with higher CBCL Externalizing Problems scores at age 3; however, our study, with its larger sample size, failed to replicate this finding. In contrast, the MIREC study results suggested potentially protective associations between prenatal exposure to PFOA and several measures of childhood neurobehavioral and social development, including the Behavior Assessment System for Children-2 (BASC-2) and the Social Responsiveness Scale-2 (SRS-2), particularly among males (Saha et al., Jan 2025). Other pregnancy cohorts, both from the U.S. and abroad, have also reported mixed results at exposure levels higher than those observed in the ECHO Cohort study sites in the present study. For example, the HOME study (N = 241) in Cincinnati, Ohio and the Shanghai-Minhang Birth Cohort Study (N = 614 preschool-age children; N = 449 school-age children) in China have reported positive associations of PFOS, PFNA, and overall PFAS mixtures with childhood behavioral problems as assessed using the CBCL and other instruments (Vuong et al., Apr 2021; Xie et al., 2023). In contrast, the Project Viva cohort (N = 950) of eastern Massachusetts, which also reported higher PFAS exposures, found no associations between prenatal PFAS exposures and childhood behavioral issues as measured by the SDQ (Harris et al., Nov 2021). The SDQ is reported to be effective at detecting inattention and hyperactivity and is at least as good as the CBCL in identifying internalizing and externalizing problems (Goodman and Scott, Feb 1999). Whether these inconsistencies can be explained by differences in prenatal exposure levels, timing of maternal serum collection, childhood behavioral instruments administered, study population racial and ethnic diversity, geographic region, measurement error, bias, unmeasured confounding or some combination thereof remains to be determined; therefore, these results should be interpreted with caution.

Despite the relatively large sample size, the current study had some limitations. While the study population consisted of a geographically diverse sample of children in the U.S., the plurality of child participants had non-Hispanic White and educated mothers due to the inherent demographic makeup of the study site samples and due to our analytic decision to drop those missing data on relevant covariates. For example, compared with children who were included in our analysis, the preschool-aged children who were excluded (n = 218) were slightly younger (mean age 3.3 vs. 3.9 years), whereas the school-aged children who were excluded (n = 46) were more likely to have younger mothers, Hispanic mothers, and mothers with a lower income. As a result, the findings may be subject to selection bias and generalizability may be limited. There was also a high level of missingness for important covariates, such as income, that were not able to be considered in the main models. Furthermore, as in any observational study, we cannot rule out the possibility of unmeasured or residual confounding. For example, data were not available for certain factors that may influence both PFAS exposure and neurodevelopment, such as maternal diet during pregnancy (Eick et al., Jan 2023; Mahmassani et al., 2022; Zheng et al., Mar 2024). Additionally, it was not possible to assess the role of postnatal PFAS exposures, alone or in combination with prenatal PFAS exposures, due to a lack of child biospecimens. Another limitation was the reliance on the CBCL for ascertaining behavioral problems. Although the CBCL is widely regarded as a highly reliable and valid assessment tool (Ivanova et al., 2007; Achenbach et al., Mar 1987; Achenbach and Edelbrock, 1981), new research suggests it may be biased by certain demographic factors, including the caregiver respondent’s primary language, education level, and sex, as well as the child’s age and race (Zheng et al., Mar 2024). Therefore, future studies of prenatal PFAS exposures with CBCL scores conducted in diverse samples should consider calculating more refined CBCL scores using checklist items identified as ‘robust’ to bias (Zheng et al., Mar 2024). Lastly, data were combined for children ages 6 through 15 whose behavior was assessed using the CBCL/6–18, but behavior problems could manifest differently within such a wide age range, particularly due to the influence of puberty (Marceau et al., Sep 2011). Due to the relatively modest number of children in this subset and the lack of complete data on age at pubertal onset, we were underpowered to conduct further analyses stratified by developmental stage (e.g., childhood, early adolescence, late adolescence). Furthermore, as pubertal timing may itself be affected by exposure to endocrine-disrupting chemicals, differences in pubertal status across participants may reflect both biological variability and environmental influences, complicating the interpretation of developmental stage as a moderator. It may also be that prenatal PFAS effects emerge during adolescence, but our analyses were underpowered to detect them. For instance, in the PELAGIE mother–child cohort (n = 444, France), prenatal exposure to PFAS were ascertained using cord serum samples and behavior was assessed using the SDQ at the age of 12 years (Tillaut et al., Nov 2023). PFOA and PFNA were found to be associated with externalizing behavioral problems, specifically hyperactivity, whereas PFNA and PFDA were associated with internalizing behavioral problems, specifically general anxiety and major depressive disorder (Tillaut et al., Nov 2023). Our study included few adolescents, which perhaps explains why most of our results were null. Future studies should consider oversampling adolescents to determine if adolescence is a sensitive period for detecting behavioral problems attributable to prenatal PFAS exposures.

Nonetheless, the present study has several notable strengths. First, we used objective measurements of PFAS exposure in serum samples collected during pregnancy. In addition, behavioral problems were ascertained using the CBCL, which is a highly reliable, consistent, and valid neurodevelopmental assessment screening tool across diverse cultures (Ivanova et al., 2007; Achenbach et al., Mar 1987; Achenbach and Edelbrock, 1981). Although the CBCL is not intended to be used as a diagnostic tool, studies have shown that it can reasonably predict some conditions, including (ADHD) and anxiety/depression (Aebi et al., Mar 2010; Biederman et al., Oct 2005). Second, as a quantitative measure of childhood behavior, the CBCL enhanced our statistical power to detect subtle associations. Third, we had a large sample size for our analysis, with children from nine study sites across seven states in the U.S. This geographic diversity also allowed us to capture a wide range of PFAS exposures as research has shown that exposures may vary by geographic location (DeLuca et al., Sep 2023).

In summary, we did not observe consistent associations between prenatal exposure to individual PFAS, or mixtures of PFAS, and altered child behavior. Our study adds to the growing body of research on prenatal PFAS exposures and neurological outcomes. Although animal studies on the topic demonstrate significant effects, the epidemiologic literature is mixed. Future research should consider assessing exposures during the postnatal period, which may be a more relevant window for childhood neurodevelopment and behavior. Additionally, as legacy PFAS are being phased out and replacements are emerging (e.g., GenX), epidemiologic studies should clarify the effects of exposures to these newer replacement chemicals. Ultimately, such knowledge is crucial for developing policies and interventions to reduce harmful environmental chemical exposures and promote optimal neurodevelopment in children.

## Supplementary Material

1

2

Supplementary data to this article can be found online at https://doi.org/10.1016/j.envint.2025.109760.

## Figures and Tables

**Table 1:** Characteristics of mothers with prenatal PFAS measured in serum and characteristics of children with a preschool- or school-age CBCL assessment.

	Preschool-Age subset (1.9–5.9 years)			School-Age subset (6–15 years)		
	Overall (n = 1,723)	Male (n = 880)	Female (n = 843)	Overall (n = 627)	Male (n = 313)	Female (n = 314)

**Child characteristic**						
Age at CBCL assessment (years), mean (SD)	3.7 (0.9)	3.7 (0.9)	3.7 (0.9)	9.4 (2.1)	9.2 (2.1)	9.5 (2.2)
**Maternal characteristic**						
Age at delivery (years), mean (SD)	29.3 (5.7)	29.5 (5.6)	29.2 (5.8)	28.0 (5.7)	28.2 (5.5)	27.8 (5.8)
Race and ethnicity*, N (%)						
Non-Hispanic White	761 (44 %)	396 (45 %)	365 (43 %)	302 (48 %)	149 (48 %)	153 (49 %)
Non-Hispanic Black	509 (30 %)	268 (30 %)	241 (29 %)	215 (34 %)	116 (37 %)	99 (32 %)
Non-Hispanic Asian	92 (5.3 %)	47 (5.3 %)	45 (5.4 %)	15 (2.4 %)	5 (1.6 %)	10 (3.2 %)
Non-Hispanic Other Race	70 (4.1 %)	36 (4.1 %)	34 (4.0 %)	33 (5.3 %)	11 (3.5 %)	22 (7.0 %)
Hispanic	288 (17 %)	132 (15 %)	156 (19 %)	62 (9.9 %)	32 (10 %)	30 (9.6 %)
Missing	<5	<5	<5	0	0	0
Educational attainment, N (%)						
Less than high school	198 (12 %)	85 (9.8 %)	113 (14 %)	70 (11 %)	26 (8.4 %)	44 (14 %)
High school diploma, GED, or equivalent	394 (23 %)	202 (23 %)	192 (23 %)	169 (27 %)	86 (28 %)	83 (27 %)
At least some college or more	1,102 (65 %)	580 (67 %)	522 (63 %)	378 (61 %)	197 (64 %)	181 (59 %)
Missing	29	13	16	10	<5	6
Annual household income, N (%)						
<$30,000	458 (40 %)	226 (39 %)	232 (41 %)	200 (62 %)	99 (61 %)	101 (62 %)
$30,000-$49,999	107 (9.3 %)	55 (9.4 %)	52 (9.2 %)	13 (4.0 %)	8 (5.0 %)	5 (3.0 %)
$50,000-$74,999	123 (11 %)	69 (12 %)	54 (9.5 %)	33 (10 %)	19 (12 %)	14 (8.5 %)
$75,000 or more	466 (40 %)	236 (40 %)	230 (40 %)	79 (24 %)	35 (22 %)	44 (27 %)
Missing	569	294	275	302	152	150
Pre-pregnancy BMI (kg/m^2^), mean (SD)	27.1 (7.4)	27.1 (7.6)	27.1 (7.1)	27.0 (8.1)	26.7 (8.4)	27.4 (7.7)
Missing	167	95	72	21	9	12
Smoking during pregnancy, N (%)						
Yes	74 (6.5 %)	37 (6.3 %)	37 (6.7 %)	22 (7.1 %)	13 (7.9 %)	9 (6.1 %)
No	1066 (93.5 %)	553 (93.7 %)	513 (93.3 %)	290 (92.9 %)	165 (92.1 %)	139 (93.9 %)
Missing	583	290	293	315	149	166
Parity, N (%)						
Parous	856 (51 %)	451 (53 %)	405 (50 %)	333 (53 %)	170 (54 %)	163 (52 %)
Nulliparous	817 (49 %)	406 (47 %)	411 (50 %)	294 (47 %)	143 (46 %)	151 (48 %)
Missing	50	23	27	0	0	0
Marital status, N (%)						
Married or living with a partner	1,164 (69 %)	592 (69 %)	572 (70 %)	417 (70 %)	209 (69 %)	208 (71 %)
Single/Separated	515 (31 %)	270 (31 %)	245 (30 %)	183 (30 %)	96 (31 %)	87 (29 %)
Missing	44	18	26	27	8	21

* Information missing on < 5 participants; actual number not provided due to confidentiality guidelines.

BMI, body mass index; CBCL, Child Behavior Checklist; GED, General Educational Development; PFAS, per- and polyfluoroalkyl substances.

**Table 2:** Summary statistics of prenatal maternal PFAS concentrations (ng/mL) detected at > 75 % frequency in serum, overall and by child sex, among children with a preschool- or school-age CBCL assessment.

			Preschool-Age subset (N = 1,723)									School-Age subset (N = 627)								
			
PFAS	LOD	Group	N	DF	p25	Median	p75	GM	GSD	Max	p_heterogeneity_	N	DF	p25	Median	p75	GM	GSD	Max	p_heterogeneity_

PFHxS	0.01–0.25	Male	880	99 %	0.6	1.1	2.1	1.1	2.7	15	0.91	313	98 %	0.7	1.6	3.1	1.4	2.7	15	0.29
		Female	843	99 %	0.5	1.1	2.2	1.0	2.8	24		314	98 %	0.8	1.8	3.1	1.5	2.6	24	
		Overall	1723	99 %	0.5	1.1	2.1	1.0	2.7	24		627	98 %	0.7	1.7	3.1	1.4	2.6	24	
PFOS	0.02–1.00	Male	880	98 %	1.5	2.5	4.2	2.4	2.6	32	0.29	313	99 %	2.5	4.1	6.5	3.9	2.3	32	0.04
		Female	843	99 %	1.4	2.5	4.7	2.6	2.5	47		314	99 %	2.5	4.9	7.8	4.4	2.2	25	
		Overall	1723	98 %	1.4	2.5	4.4	2.5	2.5	47		627	99 %	2.5	4.5	7.4	4.1	2.2	32	
PFNA	0.02–0.25	Male	880	96 %	0.2	0.3	0.5	0.3	2.6	4.5	0.04	313	99 %	0.3	0.5	0.8	0.5	2.0	4.5	0.10
		Female	843	95 %	0.2	0.4	0.6	0.3	2.8	2.3		314	99 %	0.4	0.6	0.8	0.5	1.8	2.3	
		Overall	1723	96 %	0.2	0.3	0.6	0.3	2.7	4.5		627	99 %	0.4	0.5	0.8	0.5	1.9	4.5	
PFOA	0.02–0.25	Male	880	80 %	0.4	0.8	1.3	0.7	2.7	16	0.07	313	64 %	0.8	1.2	1.8	1.1	2.0	16	0.05
		Female	843	80 %	0.5	0.9	1.4	0.7	3.0	17		314	67 %	0.9	1.3	2.0	1.3	1.9	6.3	
		Overall	1723	80 %	0.5	0.9	1.4	0.7	2.9	17		627	66 %	0.9	1.3	1.9	1.2	2.0	16	

CBCL, Child Behavior Checklist; DF, detection frequency; GM, geometric mean; GSD, geometric standard deviation; LOD, limit of detection; Max, maximum; p25, 25th percentile; p75, 75th percentile; PFOA, perfluorooctanoic acid; PFAS, per- and polyfluoroalkyl substances; PFHxS, perfluorohexane sulfonate; PFNA, perfluorononanoic acid; PFOS, perfluorooctane sulfonic acid; p_heterogeneity_, p-value comparing PFAS concentrations between males and females using Wilcoxon rank sum tests.

**Table 3:** Median (interquartile range) CBCL scores, overall and by child sex, among preschool- and school-age children with prenatal maternal PFAS measured in serum.

	Preschool-Age subset (1.9–5.9 years)				School-Age subset (6–15 years)			

	Overall (N = 1,723)	Male (N = 880)	Female (N = 843)	p_heterogeneity_	Overall (N = 627)	Male (N = 313)	Female (N = 314)	p_heterogeneity_
**Normalized T-score**								
Internalizing Problems	45 (37, 51)	45 (37, 53)	43 (37, 51)	0.11	48 (40, 53.5)	48 (41, 54)	46 (39, 52)	0.27
Emotionally Reactive	50 (50, 55)	50 (50, 55)	50 (50, 51)	0.10	-	-	-	-
Anxious/Depressed	50 (50, 52)	50 (50, 52)	50 (50, 52)	0.61	50 (50, 53)	50 (50, 53)	50 (50, 52)	0.36
Somatic Complaints	50 (50, 53)	50 (50, 53)	50 (50, 53)	0.44	53 (50, 57)	53 (50, 57)	53 (50, 57)	0.64
Withdrawn	51 (50, 56)	51 (50, 56)	51 (50, 56)	<0.01	50 (50, 54)	50 (50, 54)	50.5 (50, 54)	0.87
Externalizing Problems	44 (37, 51)	44 (39, 52)	43 (37, 50)	<0.01	46 (40, 53)	46 (40, 54)	44 (40, 51)	0.18
Attention	51 (50, 53)	51 (50, 53)	50 (50, 53)	<0.01	-	-	-	-
Aggressive Behavior	50 (50, 51)	50 (50, 52)	50 (50, 51)	0.01	50 (50, 53)	50 (50, 53)	50 (50, 52)	<0.01
Rule-Breaking Behavior	-	-	-	-	51 (50, 53)	51 (50, 53)	50 (50, 54)	0.04
Sleep Problems	51 (50, 53)	51 (50, 53)	50 (50, 53)	0.87	52 (50, 55)	52 (50, 57)	52 (50, 55)	0.33
Social Problems	-	-	-	-	51 (50, 54)	51 (50, 54)	51 (50, 54)	0.37
Thought Problems	-	-	-	-	51 (50, 54)	51 (50, 54)	50 (50, 54)	0.33
Total Problems	43 (37, 52)	44 (37, 53)	43 (37, 51)	0.01	46 (39, 52)	46 (41, 53)	46 (38, 52)	0.06
**Raw score**								
Internalizing Problems	5 (2, 8)	5 (2, 9)	4 (2, 8)	0.11	3 (1, 6)	3 (1, 6)	3 (1, 6)	0.07
Emotionally Reactive	1 (0, 2.5)	1 (0, 3)	1 (0, 2)	0.09	-	-	-	-
Anxious/Depressed	1 (0, 3)	1 (0, 3)	1 (0, 3)	0.61	1 (0, 3)	1 (0, 3)	1 (0, 3)	0.22
Somatic Complaints	1 (0, 2)	1 (0, 2)	1 (0, 2)	0.22	1 (0, 2)	1 (0, 2)	1 (0, 2)	0.39
Withdrawn	1 (0, 2)	1 (0, 2)	1 (0, 2)	<0.01	0 (0, 1)	0 (0, 1)	0.5 (0, 2)	0.11
Externalizing Problems	8 (3, 13)	8 (4, 14)	7 (3, 12)	<0.01	3 (1, 7)	3 (1, 8)	2 (1, 5)	<0.01
Attention	2 (0, 3)	2 (0.25, 3)	1 (0, 3)	<0.01	-	-	-	-
Aggressive Behavior	6 (2, 10)	6 (3, 11)	5 (2, 10)	<0.01	2 (0, 5)	2 (1, 5)	2 (0, 4)	0.01
Rule-Breaking Behavior	-	-	-	-	1 (0, 2)	1 (0, 2)	0 (0, 2)	<0.01
Sleep Problems	2 (0, 4)	2 (0, 4)	2 (0, 4)	0.82	2 (0, 5)	3 (1, 6)	2 (0, 4)	<0.01
Social Problems	-	-	-	-	1 (0, 2)	1 (0, 2)	1 (0, 2)	0.80
Thought Problems	-	-	-	-	1 (0, 2)	1 (0, 2)	0 (0, 2)	0.20
Total Problems	20 (10, 34)	20 (11, 36)	19 (9, 32)	0.01	14 (6, 23)	14 (8, 25)	13 (5, 22)	0.02

CBCL, Child Behavior Checklist; p_heterogeneity,_ p-value comparing Child Behavior Checklist scores between males and females using Wilcoxon rank sum test.

**Table 4:** Adjusted differences in CBCL scores per quartile increase in concentrations of a mixture of PFOA, PFOS, PFHxS, and PFNA in prenatal maternal serum^a,b^.

	Preschool-Age subset (N = 1,505)							School-Age subset (N = 581)					
CBCL Scores	Overall Mixture			Individual PFAS Weights				Overall Mixture			Individual PFAS Weights		
T-score	Psi^c^	95 % CI	*P*-value	PFHxS	PFOS	PFNA	PFOA	Psi^c^	95 % CI	*P*-value	PFHxS	PFOS	PFNA

Internalizing Problems	0.20	−0.58, 0.98	0.61	0.30	0.43	−1.00	0.28	−0.25	−1.32, 0.82	0.65	0.16	0.84	−1.00
Externalizing Problems	−0.19	−0.95, 0.58	0.63	0.52	0.39	−1.00	0.10	0.64	−0.47, 1.75	0.26	0.08	0.92	−1.00
Total Problems	−0.06	−0.84, 0.73	0.89	0.43	0.38	−1.00	0.19	0.06	−1.15, 1.28	0.92	−0.16	1.00	−0.84
**Raw score**													
Internalizing Problems	−0.03	−0.49, 0.42	0.88	0.75	0.25	−0.82	−0.18	−0.12	−0.63, 0.38	0.63	1.00	−0.10	−0.91
Externalizing Problems	−0.29	−0.85, 0.27	0.31	0.90	0.10	−0.52	−0.48	0.20	−0.43, 0.83	0.53	0.19	0.81	−1.00
Total Problems	−0.53	−1.97, 0.91	0.47	0.92	0.08	−0.69	−0.31	−0.14	−2.02, 1.73	0.88	0.23	0.77	−1.00

a Only PFAS biomarkers detected in more than 75% of samples in the respective subset were included. For the preschool-age subset, the mixture included PFOA, PFOS, PFNA, and PFHxS. For the school-age subset, the mixture included PFOS, PFNA, and PFHxS.

b All models were adjusted for child sex, child age at CBCL assessment, birth year, maternal age at delivery, maternal race and ethnicity, maternal pre-pregnancy BMI, maternal educational attainment, parity, marital status, and ECHO Cohort study site.

c Psi indicates overall effect estimate of PFAS mixture.

BMI, body mass index; CBCL, Child Behavior Checklist; PFAS, per- and polyfluoroalkyl substances; PFHxS, perfluorohexane sulfonate; PFNA, perfluorononanoic acid; PFOA, perfluorooctanoic acid; PFOS, perfluorooctane sulfonic acid.

## Data Availability

Select de-identified data from the ECHO Program are available through NICHD’s Data and Specimen Hub (DASH) (https://dash.nichd.nih.gov/).
