# Supplementary material for: Prenatal exposure to per- and polyfluoroalkyl substances: Association with child behavior in the environmental influences on child health outcomes (ECHO) Cohort
Source: Environ Int. Author manuscript; Available in PMC 2026 Mar 16. (PMC12989968; doi:10.1016/j.envint.2025.109760)
Supplement: 1 [file NIHMS2112776-supplement-1.docx]

**SUPPLEMENTAL MATERIAL**

**Prenatal Exposure to Per-and Polyfluoroalkyl Substances: Association with Child Behavior in the Environmental influences on Child Health Outcomes (ECHO) Cohort**

Catherine M. Bulka^a^, Lesliam Quiros-Alcala^b^, Xiaoshuang Xun^c^, T. Michael O’Shea^d^, Joseph M. Braun^e,f^, Jennifer L. Ames,^g^ Alison E. Hipwell^h^, Vaia Lida Chatzi^i^, Amy M. Padula^j^, Dana Dabelea^k,l^, Anne Starling^k,l,m^, Anne L. Dunlop^n^, Donghai Liang^o,p^, Susan Schantz^q^, Hyeong-Moo Shin^r^, Jiwon Oh^s^, Rebecca J. Schmidt^s^, Kun Lu^t^, Thomas G. O'Connor^u^, and Rebecca C. Fry^t,v^, for the ECHO Cohort Consortium*

^a^College of Public Health, University of South Florida, Tampa, FL, USA

^b^Department of Environmental Health and Engineering, Bloomberg School of Public Health, Johns Hopkins University, Baltimore, MD, USA

^c^Environmental influences on Child Health Outcomes Data Analysis Center, Johns Hopkins University, Baltimore, MD, USA

^d^Department of Pediatrics, University of North Carolina School of Medicine, Chapel Hill, NC, USA

^e^Department of Epidemiology, Brown University, Providence, RI, USA

^f^Center for Children’s Environmental Health, Brown University, Providence, RI, USA

^g^Kaiser Permanente Division of Research, Oakland, CA, USA

^h^Department of Psychiatry, University of Pittsburgh, Pittsburgh, PA, USA

^i^Department of Population and Public Health Sciences, Keck School of Medicine, University of Southern California, Los Angeles, CA, USA

^j^Program on Reproductive Health and the Environment, Department of Obstetrics, Gynecology, and Reproductive Sciences, University of California, San Francisco

^k^Lifecourse Epidemiology of Adiposity and Diabetes (LEAD) Center, University of Colorado Anschutz Medical Campus, Aurora, CO, USA

^l^Department of Epidemiology, Colorado School of Public Health, University of Colorado Anschutz Medical Campus, Aurora, CO, USA

^m^Department of Epidemiology, Gillings School of Global Public Health, University of North Carolina at Chapel Hill, Chapel Hill, NC, USA

^n^Department of Gynecology & Obstetrics, School of Medicine, Emory University, Atlanta, GA, USA

^o^Department of Environmental Health, Rollins School of Public Health, Emory University Atlanta, GA, USA

^p^Department of Epidemiology, Rollins School of Public Health, Emory University Atlanta, GA, USA

^q^Department of Psychology, College of Liberal Arts & Sciences, University of Illinois Urbana-Champaign, Champaign, IL, USA

^r^Department of Environmental Science, College of Arts & Sciences, Baylor University, Waco, TX, USA

^s^Department of Public Health Sciences, University of California Davis, Davis, California, USA

^t^Department of Environmental Sciences and Engineering, Gillings School of Global Public Health, The University of North Carolina, Chapel Hill, NC, USA

^u^Department of Psychiatry, University of Rochester Medical Center, Rochester, NY, USA

^v^Institute for Environmental Health Solutions, Gillings School of Global Public Health, The University of North Carolina, Chapel Hill, NC, USA

*See Appendix for full list of collaborators

**Corresponding Author:** Rebecca C. Fry, 135 Dauer Drive, 166A Rosenau Hall, Campus Box #7431, Chapel Hill, NC 27599-7431; 919-843-6864 (phone); [rfry@unc.edu](mailto:rfry@unc.edu) (email)

**Table of Contents**

| **Table S1.** Characteristics of nine ECHO Cohort study sites and PFAS measurements in maternal serum samples collected during pregnancy | **4** |
| --- | --- |
| **Table S2.** PFAS detected at >75% in serum with repeated measurements throughout pregnancy and intraclass correlation coefficients | **7** |
| **Table S3.** Characteristics of mothers and children included and excluded from the final analytic samples | **8** |
| **Table S4.** Summary statistics of prenatal PFAS concentrations (ng/mL) detected at less than 75% frequency in maternal serum samples, overall and by child sex, among children who completed the preschool-age or school-age CBCL assessment | **10** |
| **Table S5.** Prenatal maternal serum concentrations of legacy PFAS (ng/mL) in other epidemiologic cohort studies of childhood behavior | **11** |
| **Table S6.** Nonlinearity p-values for select adjusted associations of maternal prenatal serum PFHxS and PFOS with normalized CBCL T-scores among preschool- and school-age children | **12** |
| **Table S7.** Adjusted* mean differences (with 95% confidence intervals) in CBCL scores for a 1-ng/mL increase in PFAS detected at >75% frequency in maternal prenatal serum among preschool- and school-age children | **13** |
| **Table S8.** Adjusted associations between PFAS detected at >75% frequency in prenatal maternal serum samples and raw CBCL scores among preschool-age and school-age children, before and after adjustment for maternal smoking during pregnancy^a^ | **15** |
| **Table S9.** Interaction p-values for sex differences in adjusted associations between PFAS detected at >75% frequency in prenatal maternal serum and raw CBCL scores among preschool-age and school-age children^a,b^ | **17** |
| **Figure S1.** Directed acyclic graph depicting the hypothesized relationships among prenatal maternal PFAS exposure, childhood behavioral problems, and covariates | **18** |
| **Figure S2.** Flow diagram of inclusion and exclusion criteria used to define the final analytic samples | **19** |
| **Figure S3.** Associations between PFAS detected at >75% frequency in prenatal maternal serum samples and raw CBCL scores among preschool-age children (1.9-5 years of age) after adjustment for child sex, child age at CBCL assessment, birth year, maternal age at delivery, maternal race and ethnicity, maternal pre-pregnancy BMI, maternal educational attainment, parity, marital status, and cohort site (N=1,505) | **20** |
| **Figure S4.** Associations of PFOA and PFNA in second-trimester maternal serum samples with normalized CBCL T-scores among preschool-age children (1.9-5 years of age) after adjustment for child sex, child age at CBCL assessment, birth year, maternal age at delivery, maternal race and ethnicity, maternal pre-pregnancy BMI, maternal educational attainment, parity, marital status, and cohort site (N=953) | **21** |
| **Figure S5.** Associations between PFAS detected at <75% frequency in prenatal maternal serum samples and CBCL T-scores among preschool-age children (1.9-5 years of age) after adjustment for child sex, child age at CBCL assessment, birth year, maternal age at delivery, maternal race and ethnicity, maternal pre-pregnancy BMI, maternal educational attainment, parity, marital status, and cohort site (N=1,505) | **22** |
| **Figure S6.** Associations between PFAS detected at <75% frequency in prenatal maternal serum samples and CBCL raw scores among preschool-age children (1.9-5 years of age) after adjustment for child sex, child age at CBCL assessment, birth year, maternal age at delivery, maternal race and ethnicity, maternal pre-pregnancy BMI, maternal educational attainment, parity, marital status, and cohort site (N=1,505) | **23** |
|  |  |
| **Figure S7.** Associations between PFAS detected at >75% frequency in prenatal maternal serum samples and raw CBCL scores among school-age children (6-15 years of age) after adjustment for child sex, child age at CBCL assessment, birth year, maternal age at delivery, maternal race and ethnicity, maternal pre-pregnancy BMI, maternal educational attainment, parity, marital status, and cohort site (N=581) | **24** |
| **Figure S8.** Associations of PFOA and PFNA in second-trimester maternal serum samples with normalized CBCL T-scores among school-age children (6-15 years of age) after adjustment for child sex, child age at CBCL assessment, birth year, maternal age at delivery, maternal race and ethnicity, maternal pre-pregnancy BMI, maternal educational attainment, parity, marital status, and cohort site (N=391) | **25** |
| **Figure S9.** Associations between PFAS detected at <75% frequency in prenatal maternal serum samples and CBCL T-scores among school-age children (6-15 years of age) after adjustment for child sex, child age at CBCL assessment, birth year, maternal age at delivery, maternal race and ethnicity, maternal pre-pregnancy BMI, maternal educational attainment, parity, marital status, and cohort site (N=581) | **26** |
| **Figure S10.** Associations between PFAS detected at <75% frequency in prenatal maternal serum samples and CBCL raw scores among school-age children (6-15 years of age) after adjustment for child sex, child age at CBCL assessment, birth year, maternal age at delivery, maternal race and ethnicity, maternal pre-pregnancy BMI, maternal educational attainment, parity, marital status, and cohort site (N=581) | **27** |
| **Figure S11.** Leave-one-out analysis for prenatal PFHxS quartiles and select CBCL T-scores among the preschool-age subset across the nine ECHO Cohort study sites | **28** |
| **Figure S12.** Leave-one-out analysis for prenatal PFOS quartiles and externalizing problems CBCL T-scores among the school-age subset across the six ECHO Cohort study sites | **29** |
| **Figure S13.** Leave-one-out analysis for prenatal PFOA quartiles and select CBCL T-scores among the school-age subset across the six ECHO Cohort study sites | **30** |

| **Table S1.** Characteristics of nine ECHO Cohort study sites and PFAS measurements in maternal serum samples collected during pregnancy | | | | | | | | | |
| --- | --- | --- | --- | --- | --- | --- | --- | --- | --- |
|  | **Preschool-Age** | | **School-Age** | |  |  |  |  |  |
| **Site** | **N** | **Age range (years)** | **N** | **Age range (years)** | **State** | **Birth years** | **PFAS measurements** | **Timing of collection** | **Laboratory** |
| CiOB | 159 | 3-4.3 | 13 | 7 | California | 2014-2019 | EtFOSAA, NMFOSAA/MeFOSAA, PFBS, PFDA, PFDODA/PFDoA, PFHPA , PFHXS, PFNA, PFOA , PFOS, PFOSA, PFUNDA (N=12) | 2^nd^ and 3^rd^ trimesters | California Department of Toxic Substances Control |
|  |  |  |  |  |  |  |  |  |  |
| IKIDS | 317 | 2-5 | 50 | 6-11 | Illinois | 2014-2020 | PFOS, PFOSA, PFUNDA, PFOA, EtFOSAA, NMFOSAA/MeFOSAA, PFBS, PFDA, PFDODA/PFDoA, PFHPA , PFHXS, PFNA (N=12) | 2^nd^ trimester | California Department of Toxic Substances Control |
|  |  |  |  |  |  |  |  |  |  |
| Magee | 103 | 1.9-4.9 | 0 | - | Pennsylvania | 2017-2020 | NMFOSAA/MeFOSAA, PFBS, PFOSA, PFUNDA, PFDS, PFHPA, PFDA, PFDODA/PFDoA, PFNA, PFOA, PFHXA, PFHXS, PFOS (N=13) | 2^nd^ and 3^rd^ trimester | Human Health Exposure Analysis Resource |
|  |  |  |  |  |  |  |  |  |  |
| MADRES | 176 | 2-5 | 0 | - | California | 2016-2019 | PFNA, PFOA, PFOS, PFOSA, NMFOSAA/MeFOSAA, PFBS, PFDA, PFHPA, PFHXS, PFUNDA (N=10) | 1^st^, 2^nd^, and 3^rd^ trimester* | Human Health Exposure Analysis Resource |
|  |  |  |  |  |  |  |  |  |  |
| Atlanta ECHO | 237 | 2-5 | 37 | 6-7 | Georgia | 2014-2018 | EtFOSAA, NMFOSAA/MeFOSAA, PFBS, PFPEA, PFUNDA, PFHPA, PFHXA, PFDA, PFDODA/PFDoA, PFOA , PFOS, PFHXS, PFNA, PFOSA (N=14) | 1^st^ and 2^nd^ trimesters | Human Health Exposure Analysis Resource |
|  |  |  |  |  |  |  |  |  |  |
| CANDLE | 278 | 2.9-5.9 | 225 | 8-15 | Tennessee | 2007-2011 | PFBS, PFDA, PFDODA/PFDoA, NMFOSAA/MeFOSAA, PFOA, PFOS, PFOSA, PFUNDA, PFDS, PFHPA, PFHXA, PFHXS, PFNA, PFBA, PFPEA (N=15) | 2^nd^ and 3^rd^ trimesters | Human Health Exposure Analysis Resource |
|  |  |  |  |  |  |  |  |  |  |
| Healthy Start | 277 | 4-5 | 214 | 6-11 | Colorado | 2010-2014 | n-PFOS, NMFOSAA/MeFOSAA, PFDA, PFHXS, EtFOSAA, PFOSA, Sb-PFOA, Sm-PFOS, PFNA, n-PFOA (N=10) | 2^nd^ and 3^rd^ trimesters | Centers for Disease Control and Prevention |
|  |  |  |  |  |  |  |  |  |  |
| ARCH | 105 | 3-5 | 88 | 10-12 | Michigan | 2009-2017 | PFDA, PFHPA, PFHXA, PFDODA or PFDoA, PFDS, NMFOSAA/MeFOSAA, PFBS, PFOS, PFHXS, PFNA, PFOA, PFOSA, PFUNDA (N=13) | 1^st^, 2^nd^, and 3^rd^ trimesters* | Human Health Exposure Analysis Resource |
|  |  |  |  |  |  |  |  |  |  |
| PETALS | 71 | 3.8-5.9 | 0 | - | California | 2014-2017 | EtFOSAA, PFHPA, PFHXA, PFHXS, NMFOSAA/MeFOSAA, PFBS, PFDA, PFDODA/PFDoA, PFPEA, PFUNDA, PFNA, PFOA, PFOS, PFOSA (N=14) | 1^st^ and 2^nd^ trimesters | Human Health Exposure Analysis Resource |

ECHO, Environmental influences on Child Health Outcomes; PFAS, per- and polyfluoroalkyl substances.

*Note that no one individual had serum samples collected in all three trimesters.

**Table S2.** PFAS detected at >75% in serum with repeated measurements throughout pregnancy and intraclass correlation coefficients

|  | **1^st^ and 2^nd^ trimester** | | **2^nd^ and 3^rd^ trimester** | | **1^st^ and 3^rd^ trimester** | |
| --- | --- | --- | --- | --- | --- | --- |
| **PFAS** | **N** | **ICC** | **N** | **ICC** | **N** | **ICC** |
| PFHXS | 69 | 0.86 | 291 | 0.84 | 5 | 0.83 |
| PFNA | 69 | 0.88 | 290 | 0.70 | 6 | 0.89 |
| PFOA | 69 | 0.92 | 291 | 0.56 | 5 | 0.28 |
| PFOS | 69 | 0.94 | 291 | 0.91 | 5 | 0.92 |

PFAS, per- and polyfluoroalkyl substances; PFHXS, perfluorohexane sulfonic acid; PFNA, perfluorononanoic acid; PFOA, perfluorooctanoic acid; PFOS, perfluorooctane sulfonic acid.

| **Table S3.** Characteristics of mothers and children included and excluded from the final analytic samples | | | | | | |  |
| --- | --- | --- | --- | --- | --- | --- | --- |
|  | **Preschool-Age subset**  **(1.9-5 years)** | | | **School-Age subset**  **(6-15 years)** | | | |
|  | **Overall**  **(N=1,723)** | **Included**  **(N=1,505)** | **Excluded**  **(N=218)** | **Overall**  **(N=627)** | **Included**  **(N=581)** | **Excluded**  **(N=46)** | |
| **Child characteristic** |  |  |  |  |  |  | |
| Age at CBCL assessment (years), mean (SD) | 3.7 (0.9) | 3.8 (0.9) | 3.3 (0.9) | 9.4 (2.1) | 9.3 (2.2) | 9.9 (1.8) | |
| **Maternal characteristic** |  |  |  |  |  |  | |
| Age at delivery (years), mean (SD) | 29.3 (5.7) | 29.3 (5.7) | 29.7 (5.6) | 28.0 (5.7) | 28.2 (5.7) | 25.5 (5.4) | |
| Race and ethnicity*, N (%) |  |  |  |  |  |  | |
| Non-Hispanic White | 761 (44%) | 658 (44%) | 103 (48%) | 302 (48%) | 284 (49%) | 18 (39%) | |
| Non-Hispanic Black | 509 (30%) | 454 (30%) | 55 (26%) | 215 (34%) | 202 (35%) | 13 (28%) | |
| Non-Hispanic Asian | 92 (5.3%) | 77 (5.1%) | <5 | 15 (2.4%) | 15 (2.6%) | 0 (0.0%) | |
| Non-Hispanic Other Race | 70 (4.1%) | 61 (4.1%) | 9 (4.2%) | 33 (5.3%) | 27 (4.6%) | 6 (13%) | |
| Hispanic | 288 (17%) | 255 (16%) | 44 (20%) | 62 (9.9%) | 53 (9.1%) | 9 (20%) | |
| Educational attainment, N (%) |  |  |  |  |  |  | |
| Less than high school | 198 (12%) | 176 (12%) | 22 (12%) | 70 (11%) | 62 (11%) | 8 (22%) | |
| High school diploma, GED, or equivalent | 394 (23%) | 354 (24%) | 40 (21%) | 169 (27%) | 158 (27%) | 11 (31%) | |
| At least some college or more | 1,102 (65%) | 975 (65%) | 127 (67%) | 378 (61%) | 361 (62%) | 17 (47%) | |
| Annual household income, N (%) |  |  |  |  |  |  | |
| <$30,000 | 458 (40%) | 383 (39%) | 75 (43%) | 200 (62%) | 171 (59%) | 29 (81%) | |
| $30,000-$49,999 | 107 (9.3%) | 90 (9.2%) | 17 (9.7%) | 13 (4.0%) | <13 | <5 | |
| $50,000-$74,999 | 123 (11%) | 102 (10%) | 21 (12%) | 33 (10%) | <33 | <5 | |
| $75,000 or more | 466 (40%) | 403 (41%) | 63 (36%) | 79 (24%) | <79 | <5 | |
| Pre-pregnancy BMI (kg/m^2^), mean (SD) | 27.1 (7.4) | 27.1 (7.4) | 29.0 (7.0) | 27.0 (8.1) | 27.1 (8.1) | 26.1 (7.2) | |
| Smoking during pregnancy, N (%) |  |  |  |  |  |  | |
| Yes | 74 (6.5%) | 67 (6.3%) | 7 (10%) | 22 (7.1%) | <5 | <22 | |
| No |  | 1,438 (95.5%) |  |  |  |  | |
| Parity, N (%) |  |  |  |  |  |  | |
| Parous | 856 (51%) | 776 (52%) | 80 (48%) | 333 (53%) | 308 (53%) | 25 (54%) | |
| Nulliparous | 817 (49%) | 729 (48%) | 88 (52%) | 294 (47%) | 273 (47%) | 21 (46%) | |
| Marital status, N (%) |  |  |  |  |  |  | |
| Married or living with a partner | 1,164 (69%) | 1,055 (70%) | 109 (63%) | 417 (70%) | <417 | <5 | |
| Single/Separated | 515 (31%) | 450 (30%) | 65 (37%) | 183 (30%) | <183 | <5 | |
| *Information missing on <5 participants; actual number not provided due to confidentiality guidelines.  BMI, body mass index; CBCL, Child Behavior Checklist; GED, General Educational Development; SD, standard deviation. | | | | | | |  |

**Table S4.** Summary statistics of prenatal PFAS concentrations (ng/mL) detected at less than 75% frequency in maternal serum samples, overall and by child sex, among children who completed the preschool-age or school-age CBCL assessment

|  |  |  |  |  | **Preschool-Age subset** | | | | | | | | **School-Age subset** | | | | | | | | |
| --- | --- | --- | --- | --- | --- | --- | --- | --- | --- | --- | --- | --- | --- | --- | --- | --- | --- | --- | --- | --- | --- |
| **PFAS** | **LODs** | **Group** | **N** | **DF** |  | **p25** | **Median** | **p75** | **GM** | **GSD** | **Max** | **p_heterogeneity_** | **N** | **DF** | **p25** | **Median** | **p75** | **GM** | **GSD** | **Max** | **p_heterogeneity_** |
| PFDA | 0.01-0.25 | Male | 829 | 57% |  | <LOD | 0.1 | 0.1 | 0.1 | 2.6 | 3.5 | 0.53 | 292 | 52% | <LOD | 0.1 | 0.2 | 0.1 | 2.7 | 3.5 | 0.80 |
|  |  | Female | 797 | 57% |  | <LOD | 0.1 | 0.1 | 0.1 | 2.5 | 2.3 |  | 294 | 53% | <LOD | 0.1 | 0.2 | 0.1 | 2.7 | 0.7 |  |
|  |  | Overall | 1626 | 57% |  | <LOD | 0.1 | 0.1 | 0.1 | 2.5 | 3.5 |  | 586 | 52% | <LOD | 0.1 | 0.2 | 0.1 | 2.7 | 3.5 |  |
| NMFOSAA/ MeFOSAA | 0.01-0.04 | Male | 825 | 53% |  | <LOD | 0.1 | 0.1 | 0.1 | 3.0 | 2.0 | 0.20 | 292 | 52% | <LOD | 0.1 | 0.1 | 0.1 | 2.7 | 2.0 | 0.11 |
|  |  | Female | 797 | 58% |  | <LOD | 0.1 | 0.1 | 0.1 | 3.2 | 2.2 |  | 295 | 57% | 0.1 | 0.1 | 0.2 | 0.1 | 2.8 | 2.2 |  |
|  |  | Overall | 1622 | 55% |  | <LOD | 0.1 | 0.1 | 0.1 | 3.1 | 2.2 |  | 587 | 55% | 0.1 | 0.1 | 0.2 | 0.1 | 2.7 | 2.2 |  |
| PFUNDA | 0.02-0.50 | Male | 699 | 52% |  | <LOD | <LOD | 0.1 | 0.1 | 3.0 | 1.9 | 0.35 | 203 | 48% | <LOD | 0.1 | 0.2 | 0.1 | 3.1 | 1.2 | 0.75 |
|  |  | Female | 682 | 54% |  | <LOD | 0.1 | 0.1 | 0.1 | 2.9 | 0.9 |  | 207 | 53% | <LOD | 0.1 | 0.2 | 0.1 | 2.7 | 0.7 |  |
|  |  | Overall | 1381 | 53% |  | <LOD | 0.1 | 0.1 | 0.1 | 3.0 | 1.9 |  | 410 | 50% | <LOD | 0.1 | 0.2 | 0.1 | 2.9 | 1.2 |  |
| PFPEA | 0.02-0.25 | Male | 150 | 49% |  | <LOD | 0.1 | 0.2 | 0.1 | 2.4 | 0.8 | 0.15 | 43 | 9% | <LOD | 0.2 | 0.2 | 0.1 | 2.2 | 0.2 | 0.48 |
|  |  | Female | 154 | 56% |  | <LOD | 0.1 | 0.2 | 0.1 | 2.5 | 0.7 |  | 35 | 6% | <LOD | 0.2 | 0.2 | 0.1 | 2.1 | 0.2 |  |
|  |  | Overall | 304 | 52% |  | <LOD | 0.1 | 0.2 | 0.1 | 2.5 | 0.8 |  | 78 | 8% | <LOD | 0.2 | 0.2 | 0.1 | 2.2 | 0.2 |  |
| PFDS | 0.02 | Male | 232 | 26% |  | <LOD | <LOD | <LOD | <LOD | 3.2 | 10 | 0.01 | 128 | 31% | <LOD | <LOD | <LOD | <LOD | 2.1 | 5.4 | 0.41 |
|  |  | Female | 216 | 22% |  | <LOD | <LOD | <LOD | <LOD | 2.0 | 6.5 |  | 148 | 26% | <LOD | <LOD | <LOD | <LOD | 1.6 | 0.1 |  |
|  |  | Overall | 448 | 24% |  | <LOD | <LOD | <LOD | <LOD | 2.7 | 10 |  | 276 | 28% | <LOD | <LOD | <LOD | <LOD | 1.9 | 5.4 |  |
| PFHxA | 0.05-0.50 | Male | 379 | 15% |  | <LOD | <LOD | <LOD | <LOD | 3.2 | 10 | 0.52 | 169 | 4% | <LOD | <LOD | <LOD | <LOD | 2.3 | 5.4 | 0.93 |
|  |  | Female | 364 | 14% |  | <LOD | <LOD | <LOD | <LOD | 2.5 | 6.5 |  | 178 | 1% | <LOD | <LOD | <LOD | <LOD | 2.0 | 0.4 |  |
|  |  | Overall | 743 | 14% |  | 0.0 | <LOD | <LOD | <LOD | 2.8 | 10 |  | 347 | 2% | <LOD | <LOD | <LOD | <LOD | 2.1 | 5.4 |  |
| PFHpA | 0.03-0.50 | Male | 678 | 12% |  | <LOD | <LOD | <LOD | <LOD | 1.9 | 0.6 | 0.07 | 201 | 6% | <LOD | <LOD | <LOD | <LOD | 2.3 | 0.6 | 0.69 |
|  |  | Female | 663 | 10% |  | <LOD | <LOD | <LOD | <LOD | 1.9 | 0.5 |  | 206 | 6% | <LOD | <LOD | <LOD | <LOD | 2.2 | 0.5 |  |
|  |  | Overall | 1341 | 11% |  | 0.0 | <LOD | <LOD | <LOD | 1.9 | 0.6 |  | 407 | 6% | <LOD | <LOD | <LOD | <LOD | 2.2 | 0.6 |  |
| PFBS | 0.02-2.50 | Male | 655 | 9% |  | <LOD | <LOD | <LOD | <LOD | 2.6 | 1.8 | 0.99 | 201 | 9% | <LOD | <LOD | <LOD | <LOD | 4.3 | 1.8 | 0.77 |
|  |  | Female | 638 | 8% |  | <LOD | <LOD | <LOD | <LOD | 2.5 | 1.8 |  | 205 | 9% | <LOD | <LOD | <LOD | <LOD | 4.0 | 1.8 |  |
|  |  | Overall | 1293 | 9% |  | <LOD | <LOD | <LOD | <LOD | 2.5 | 1.8 |  | 406 | 9% | <LOD | <LOD | <LOD | <LOD | 4.1 | 1.8 |  |
| PFBA | 0.25 | Male | 24 | 8% |  | <LOD | <LOD | <LOD | <LOD | 1.2 | 0.5 | 0.91 | 22 | 9% | <LOD | <LOD | <LOD | <LOD | 1.3 | 0.5 | 0.94 |
|  |  | Female | 23 | 9% |  | <LOD | <LOD | <LOD | <LOD | 1.5 | 0.8 |  | 22 | 9% | <LOD | <LOD | <LOD | <LOD | 1.5 | 0.8 |  |
|  |  | Overall | 27 | 9% |  | <LOD | <LOD | <LOD | <LOD | 1.4 | 0.8 |  | 44 | 9% | <LOD | <LOD | <LOD | <LOD | 1.4 | 0.8 |  |
| EtFOSAA | 0.01-0.02 | Male | 488 | 7% |  | <LOD | <LOD | 0.1 | <LOD | 2.5 | 0.2 | 0.27 | 163 | 4% | <LOD | 0.1 | 0.1 | <LOD | 2.4 | 0.1 | 0.22 |
|  |  | Female | 465 | 8% |  | <LOD | <LOD | 0.1 | <LOD | 2.5 | 0.2 |  | 146 | 5% | <LOD | 0.1 | 0.1 | <LOD | 2.4 | 0.1 |  |
|  |  | Overall | 953 | 8% |  | <LOD | <LOD | <LOD | <LOD | 2.5 | 0.2 |  | 309 | 5% | <LOD | 0.1 | 0.1 | <LOD | 2.4 | 0.1 |  |
| PFDoDA/ PFDoA | 0.04-0.20 | Male | 571 | 3% |  | <LOD | <LOD | 0.1 | <LOD | 1.9 | 0.7 | 0.23 | 180 | 3% | <LOD | <LOD | <LOD | <LOD | 1.5 | 0.1 | 0.15 |
|  |  | Female | 563 | 4% |  | <LOD | <LOD | 0.1 | <LOD | 2.0 | 1.6 |  | 189 | 7% | <LOD | <LOD | <LOD | 0.1 | 1.7 | 0.5 |  |
|  |  | Overall | 1134 | 4% |  | <LOD | <LOD | <LOD | <LOD | 2.0 | 1.6 |  | 369 | 5% | <LOD | <LOD | <LOD | <LOD | 1.6 | 0.5 |  |
| PFOSA | 0.01-0.10 | Male | 800 | 3% |  | <LOD | <LOD | <LOD | <LOD | 2.0 | 0.1 | 0.82 | 290 | 1% | <LOD | <LOD | 0.1 | <LOD | 2.2 | 0.1 | 0.77 |
|  |  | Female | 770 | 4% |  | <LOD | <LOD | <LOD | <LOD | 1.9 | 0.1 |  | 294 | 2% | <LOD | <LOD | 0.1 | <LOD | 2.2 | 0.1 |  |
|  |  | Overall | 1570 | 3% |  | <LOD | <LOD | <LOD | <LOD | 1.9 | 0.1 |  | 584 | 2% | <LOD | <LOD | 0.1 | <LOD | 2.2 | 0.1 |  |
| CBCL, Child Behavior Checklist; DF, detection frequency; EtFOSAA, N-ethyl perfluorooctane sulfonamido acetic acid; GM, geometric mean; GSD, geometric standard deviation; LOD, limit of detection; Max, maximum; NMFOSAA/MeFOSAA, N-methyl perfluorooctanesulfonamidoacetic acid; PFAS, per- and polyfluoroalkyl substances; PFBA, perfluorobutanoic acid; PFBS, perfluorobutanesulfonic acid; PFDA, perfluorodecanoic acid; PFDS, perfluorodecanesulfonic acid; PFDODA/PFDoA, perfluorododecanoic acid; PFHxA, perfluorohexanoic acid; PFHpA, perfluoroheptanoic acid; PFOSA, perfluorooctanesulfonamide; P_heterogeneity_, p-value comparing PFAS concentrations between males and females using Wilcoxon rank sum tests; p25, 25th percentile; p75, 75th percentile; PFPEA, perfluoropentanoic acid; PFUNDA, perfluoroundecanoic acid. | | | | | | | | | | | | | | | | | | | | | |

| **Table S5.** Prenatal maternal serum concentrations of legacy PFAS (ng/mL) in other epidemiologic cohort studies of childhood behavior | | | | | | | | | | | | | | | | |
| --- | --- | --- | --- | --- | --- | --- | --- | --- | --- | --- | --- | --- | --- | --- | --- | --- |
| **Author (Year)** | **Cohort** |  | **Country (State)** | **Gestational age** | **Analyte** | **N** | **% Detected** | **GM (GSD)** | **Percentile** | | | | | | | |
|  |  | **Sampling year** |  |  |  |  |  |  | **5** | **10** | **25** | **50** | **75** | **90** | **95** | **100** |
| Vuong *et al.* (2021)^13^ | Health Outcomes and Measures of the Environment (HOME) Study | 2003-2006 | USA (OH) | 16+ weeks | PFOA | 241 | 100 | 5.3 (1.7) | - | - | 3.6 | 5.4 | 7.5 | - | 12 | - |
|  |  |  |  |  | PFOS | 241 | 100 | 12.8 (1.7) | - | - | 8.8 | 13.2 | 17.8 | - | 29.4 | - |
|  |  |  |  |  | PFHxS | 241 | 99.6 | 1.5 (2.2) | - | - | 0.9 | 1.5 | 2.4 | - | 5 | - |
|  |  |  |  |  | PFNA | 240 | 100 | 0.9 (1.5) | - | - | 0.7 | 0.9 | 1.1 | - | 1.9 | - |
| Choi *et al.* (2024)^14^ | Markers of Autism Risk in Babies - Learning Early Signals (MARBLES) | 2009-2015 | USA (CA) | 1st-3rd trimester | PFOA | 280 | 100 | 0.87 (1.74) | 0.35 |  | 0.60 | 0.9 | 1.20 | - | 2.10 | - |
|  |  |  |  |  | PFOS | 280 | 100 | 2.93 (1.82) | 1.10 |  | 2.10 | 3.05 | 4.23 | - | 7.00 | - |
|  |  |  |  |  | PFHxS | 280 | 99.3 | 0.45 (1.98) | 0.20 |  | 0.30 | 0.5 | 0.70 | - | 1.60 | - |
|  |  |  |  |  | PFNA | 280 | 99.6 | 0.48 (1.67) | 0.20 |  | 0.40 | 0.5 | 0.70 | - | 1.00 | - |
| Harris *et al.* (2021)^15^ | Project Viva | 1999-2002 | USA (MA) | <22 weeks | PFOA | 950 | 100 | - | - | - | 3.9 | 5.6 | 7.7 | - | - | 49 |
|  |  |  |  |  | PFOS | 950 | 99.8 | - | - | - | 18.2 | 24.7 | 33.5 | - | - | 168 |
|  |  |  |  |  | PFHxS | 950 | 99.2 | - | - | - | 1.6 | 2.3 | 3.7 | - | - | 43 |
|  |  |  |  |  | PFNA | 950 | 98.8 | - | - | - | 0.5 | 0.6 | 0.9 | - | - | 6 |
| Xie *et al.* (2022)^16^ | Shanghai-Minhang Birth Cohort Study | 2012 | China | 12-16 weeks | PFOA | 614 | 100 | 19.82 (1.55) | 9.3 | - | 15.2 | 19.7 | 27.2 | - | 38.5 | - |
|  |  |  |  |  | PFOS | 614 | 100 | 10.82 (1.75) | 4.1 | - | 7.55 | 10.8 | 15.9 | - | 25.2 | - |
|  |  |  |  |  | PFHxS | 614 | 100 | 2.72 (1.53) | 1.4 | - | 2.1 | 2.77 | 3.54 | - | 5.65 | - |
|  |  |  |  |  | PFNA | 614 | 100 | 1.80 (1.62) | 0.8 | - | 1.32 | 1.79 | 2.49 | - | 3.98 | - |
| Xie *et al.* (2023)^17^ | Shanghai-Minhang Birth Cohort Study | 2012 | China | 12-16 weeks | PFOA | 449 | 100 | 19.96 (1.56) | 9 | - | 15.4 | 19.8 | 27.2 | - | 39.1 | - |
|  |  |  |  |  | PFOS | 449 | 100 | 11.03 (1.73) | 4.5 | - | 7.71 | 10.9 | 16.2 | - | 24.2 | - |
|  |  |  |  |  | PFHxS | 449 | 100 | 2.76 (1.55) | 1.5 | - | 2.07 | 2.78 | 3.56 | - | 5.96 | - |
|  |  |  |  |  | PFNA | 449 | 100 | 1.82 (1.63) | 0.8 | - | 1.34 | 1.81 | 2.53 | - | 3.98 | - |

GM, geometric mean; GSD, geometric standard deviation; PFAS, per- and polyfluoroalkyl substances; PFHxS, perfluorohexane sulfonic acid; PFNA, perfluorononanoic acid; PFOA, perfluorooctanoic acid; PFOS, perfluorooctane sulfonic acid.

| **Table S6.** Nonlinearity p-values for select adjusted associations of maternal prenatal serum PFHxS and PFOS with normalized CBCL T-scores among preschool- and school-age children | | | | |
| --- | --- | --- | --- | --- |
|  | **Preschool-Age subset**  **(1.9-5 years)**  **(N=1,505)** | **School-Age subset** | | |
|  |  | **(6-15 years)** | | |
|  |  | **(N=581)** | | |
| **Normalized T-scores** | **PFHxS** |  | **PFHxS** | **PFOS** |
| Internalizing Problems | **<0.01** |  | 0.08 | - |
| Emotionally Reactive | 0.08 |  | - | - |
| Anxious/Depressed | 0.17 |  | 0.38 | - |
| Somatic Complaints | 0.45 |  | 0.26 | - |
| Withdrawn | **0.03** |  | 0.38 | - |
| Externalizing Problems | 0.29 |  | 0.42 | 0.42 |
| Attention | 0.36 |  | 0.26 | - |
| Aggressive Behavior | 0.41 |  | 0.87 | - |
| Rule-Breaking Behavior | - |  | 0.66 | - |
| Sleep Problems | 0.68 |  | - | - |
| Social Problems | - |  | 0.84 | - |
| Thought Problems | - |  | **0.04** | - |
| Total Problems | **0.04** |  | 0.08 | - |
| \| CBCL, Child Behavior Checklist; PFHxS, perfluorohexane sulfonate; PFOS, perfluorooctane sulfonic acid.  **Bolded values are statistically significant at p<0.05.**  *All models were adjusted for child sex, child age at CBCL assessment, birth year, maternal age at delivery, maternal race and ethnicity, maternal pre-pregnancy body mass index (BMI), maternal educational attainment, parity, marital status, and ECHO Cohort study site. \| \| --- \| | | | | |

| **Table S7.** Adjusted* mean differences (with 95% confidence intervals) in CBCL scores for a 1-ng/mL increase in PFAS detected at >75% frequency in maternal prenatal serum among preschool- and school-age children | | | | | | | | |
| --- | --- | --- | --- | --- | --- | --- | --- | --- |
|  | **Preschool-Age subset** | | |  | **School-Age subset** | | | |
|  | **(1.9-5 years)** | | |  | **(6-15 years)** | | | |
|  | **N=1,505** | | |  | **N=581** | | | |
| **Normalized T-scores** | **PFHxS** | **PFOS** | **PFNA** | **PFOA** |  | **PFHxS** | **PFOS** | **PFNA** |
| Internalizing Problems | 0.07 (-0.32, 0.47) | -0.02 (-0.22, 0.18) | -1.05 (-2.70, 0.60) | -0.01 (-0.60, 0.58) |  | 0.18 (-0.26, 0.61) | 0.03 (-0.22, 0.26) | 0.91 (-0.98, 2.8) |
| Emotionally Reactive | 0.06 (-0.12, 0.24) | 0.00 (-0.10, 0.09) | -0.06 (-0.85, 0.74) | 0.08 (-0.20, 0.37) |  | - | - | - |
| Anxious/Depressed | -0.01 (-0.18, 0.17) | -0.05 (-0.14, 0.03) | -0.43 (-1.15, 0.28) | -0.06 (-0.32, 0.19) |  | 0.14 (-0.08, 0.35) | 0.05 (-0.06, 0.16) | 0.35 (-0.59, 1.29) |
| Somatic Complaints | 0.00 (-0.18, 0.17) | 0.01 (-0.08, 0.10) | 0.00 (-0.78, 0.79) | 0.08 (-0.20, 0.36) |  | 0.14 (-0.08, 0.35) | 0.05 (-0.06, 0.16) | 0.35 (-0.59, 1.29) |
| Withdrawn | 0.07 (-0.17, 0.30) | -0.07 (-0.19, 0.04) | 0.32 (-0.64, 1.28) | 0.26 (-0.08, 0.60) |  | -0.04 (-0.26, 0.18) | -0.03 (-0.15, 0.08) | **1.17 (0.20, 2.16)** |
| Externalizing Problems | 0.07 (-0.33, 0.46) | -0.09 (-0.28, 0.11) | -0.13 (-1.75, 1.5) | -0.14 (-0.72, 0.45) |  | 0.15 (-0.3, 0.60) | 0.12 (-0.12, 0.36) | 1.14 (-0.84, 3.12) |
| Attention | 0.08 (-0.10, 0.26) | -0.08 (-0.18, 0.01) | -0.39 (-1.20, 0.42) | -0.16 (-0.45, 0.13) |  | -0.11 (-0.39, 0.17) | -0.08 (-0.23, 0.08) | 1.01 (-0.19, 2.21) |
| Aggressive Behavior | 0.02 (-0.15, 0.18) | -0.03 (-0.12, 0.05) | -0.02 (-0.72, 0.68) | -0.05 (-0.30, 0.20) |  | 0.00 (-0.22, 0.22) | -0.01 (-0.13, 0.11) | 0.25 (-0.67, 1.17) |
| Rule-Breaking Behavior | - | - | - | - |  | 0.02 (-0.24, 0.28) | 0.00 (-0.13, 0.15) | 0.51 (-0.58, 1.59) |
| Sleep Problems | 0.2 (-0.01, 0.41) | 0.00 (-0.11, 0.11) | -0.70 (-1.64, 0.24) | -0.11 (-0.44, 0.23) |  | - | - | - |
| Social Problems | - | - | - | - |  | 0.01 (-0.22, 0.24) | -0.04 (-0.16, 0.08) | 0.00 (-0.96, 0.96) |
| Thought Problems | - | - | - | - |  | -0.03 (-0.26, 0.21) | -0.01 (-0.14, 0.12) | 1.01 (-0.03, 2.05) |
| Total Problems | 0.08 (-0.33, 0.48) | -0.08 (-0.28, 0.13) | -0.70 (-2.37, 0.97) | -0.14 (-0.74, 0.46) |  | -0.01 (-0.51, 0.50) | 0.02 (-0.26, 0.30) | 1.05 (-1.11, 3.22) |
| **Raw scores** |  |  |  |  |  |  |  |  |
| Internalizing Problems | 0.02 (-0.22, 0.26) | -0.04 (-0.16, 0.07) | -0.56 (-1.57, 0.45) | 0.00 (-0.35, 0.36) |  | 0.12 (-0.08, 0.33) | 0.03 (-0.09, 0.14) | 0.71 (-0.18, 1.60) |
| Emotionally Reactive | 0.01 (-0.07, 0.08) | -0.01 (-0.04, 0.03) | -0.16 (-0.49, 0.17) | 0.01 (-0.11, 0.12) |  | - | - | - |
| Anxious/Depressed | 0.00 (-0.08, 0.08) | -0.02 (-0.06, 0.02) | **-0.43 (-0.76, -0.10)** | -0.09 (-0.20, 0.03) |  | 0.08 (-0.03, 0.20) | 0.03 (-0.03, 0.09) | 0.16 (-0.33, 0.64) |
| Somatic Complaints | -0.02 (-0.09, 0.04) | 0.00 (-0.03, 0.03) | -0.13 (-0.41, 0.16) | -0.01 (-0.11, 0.09) |  | 0.05 (-0.03, 0.13) | 0.01 (-0.03, 0.05) | 0.23 (-0.12, 0.59) |
| Withdrawn | 0.04 (-0.04, 0.11) | -0.01 (-0.05, 0.02) | 0.14 (-0.17, 0.44) | 0.09 (-0.01, 0.20) |  | -0.02 (-0.08, 0.05) | -0.01 (-0.05, 0.03) | **0.32 (0.02, 0.62)** |
| Externalizing Problems | 0.04 (-0.24, 0.32) | -0.08 (-0.22, 0.06) | -0.51 (-1.77, 0.75) | -0.19 (-0.63, 0.25) |  | 0.01 (-0.26, 0.29) | 0.02 (-0.12, 0.17) | 0.49 (-0.65, 1.63) |
| Attention | 0.02 (-0.06, 0.09) | **-0.04 (-0.08, 0.00)** | -0.20 (-0.52, 0.11) | -0.10 (-0.22, 0.01) |  | -0.06 (-0.22, 0.10) | -0.03 (-0.11, 0.06) | 0.53 (-0.15, 1.2) |
| Aggressive Behavior | -0.02 (-0.24, 0.21) | -0.03 (-0.14, 0.08) | -0.22 (-1.20, 0.77) | -0.07 (-0.42, 0.27) |  | -0.01 (-0.09, 0.07) | 0.00 (-0.05, 0.04) | 0.11 (-0.24, 0.47) |
| Rule-Breaking Behavior | - | - | - | - |  | 0.03 (-0.17, 0.24) | 0.03 (-0.08, 0.14) | 0.37 (-0.47, 1.22) |
| Sleep Problems | 0.03 (-0.06, 0.12) | -0.01 (-0.06, 0.03) | **-0.44 (-0.84, -0.03)** | -0.10 (-0.25, 0.04) |  | - | - | - |
| Social Problems | - | - | - | - |  | 0.01 (-0.09, 0.12) | -0.02 (-0.07, 0.04) | 0.03 (-0.41, 0.47) |
| Thought Problems | 0.16 (-0.55, 0.88) | -0.20 (-0.56, 0.16) | -2.14 (-5.35, 1.08) | -0.45 (-1.57, 0.67) |  | -0.01 (-0.09, 0.08) | 0.00 (-0.05, 0.05) | 0.32 (-0.07, 0.71) |
| Total Problems | 0.07 (-0.32, 0.47) | -0.02 (-0.22, 0.18) | -1.05 (-2.70, 0.60) | -0.01 (-0.60, 0.58) |  | 0.04 (-0.74, 0.82) | -0.06 (-0.48, 0.36) | 2.08 (-1.28, 5.43) |

| CBCL, Child Behavior Checklist; PFOA, perfluorooctanoic acid; PFAS, per- and polyfluoroalkyl substances; PFHxS, perfluorohexane sulfonate; PFNA, perfluorononanoic acid; PFOS, perfluorooctane sulfonic acid. |
| --- |

**Bolded values are statistically significant at p<0.05.**

*All models were adjusted for child sex, child age at CBCL assessment, birth year, maternal age at delivery, maternal race and ethnicity, maternal pre-pregnancy body mass index (BMI), maternal educational attainment, parity, marital status, and ECHO Cohort study site

**Table S8.** Adjusted associations between PFAS detected at >75% frequency in prenatal maternal serum samples and raw CBCL scores among preschool-age and school-age children, before and after adjustment for maternal smoking during pregnancy^a^

|  |  |  | **Preschool-Age subset**  **(N=1,140)** | | | | **School-Age subset**  **(N=312)** | | | |
| --- | --- | --- | --- | --- | --- | --- | --- | --- | --- | --- |
|  |  |  | **Without adjustment** | | **With adjustment** | | **Without adjustment** | | **With adjustment** | |
| **PFAS** | **CBCL score** | **Quartile** | **Estimate (95% CI)** | **p_trend_** | **Estimate (95% CI)** | **p_trend_** | **Estimate (95% CI)** | **p_trend_** | **Estimate (95% CI)** | **p_trend_** |
| PFHxS | Internalizing Problems | 1 | 0.00 (reference) | 0.41 | 0.00 (reference) | 0.43 | 0.00 (reference) | 0.48 | 0.00 (reference) | 0.48 |
|  |  | 2 | 1.15 (0.27, 2.00) |  | 1.15 (0.25, 2.00) |  | 0.95 (-0.14, 2.03) |  | 0.95 (-0.13, 2.04) |  |
|  |  | 3 | 1.04 (0.00, 1.99) |  | 1.03 (-0.01, 1.99) |  | 1.07 (-0.42, 2.56) |  | 1.07 (-0.42, 2.57) |  |
|  |  | 4 | 0.05 (-1.38, 1.38) |  | 0.04 (-1.40, 1.38) |  | -0.25 (-2.16, 1.66) |  | -0.25 (-2.16, 1.66) |  |
|  | Externalizing Problems | 1 | 0.00 (reference) | 0.56 | 0.00 (reference) | 0.58 | 0.00 (reference) | 0.19 | 0.00 (reference) | 0.20 |
|  |  | 2 | 0.53 (-0.56, 1.61) |  | 0.48 (-0.60, 1.83) |  | 0.57 (-0.74, 1.88) |  | 0.53 (-0.78, 1.85) |  |
|  |  | 3 | 0.62 (-0.60, 1.84) |  | 0.61 (-0.60, 1.74) |  | 1.36 (-0.45, 3.16) |  | 1.33 (-0.48, 3.14) |  |
|  |  | 4 | 0.06 (-1.65, 1.76) |  | 0.04 (-1.67, 1.74) |  | 0.82 (-1.49, 3.13) |  | 0.81 (-1.50, 3.13) |  |
|  | Total Problems | 1 | 0.00 (reference) | 0.41 | 0.00 (reference) | 0.42 | 0.00 (reference) | 0.17 | 0.00 (reference) | 0.18 |
|  |  | 2 | 2.26 (-0.44, 4.97) |  | 2.16 (-0.58, 4.87) |  | 3.02 (-0.86, 6.89) |  | 3 (-0.89, 6.88) |  |
|  |  | 3 | 2.63 (-0.48, 5.68) |  | 2.61 (-0.60, 5.65) |  | 5.2 (-0.14, 10.55) |  | 5.19 (-0.16, 10.54) |  |
|  |  | 4 | -0.23 (-4.50, 4.03) |  | -0.28 (-4.67, 3.97) |  | 1.18 (-5.66, 8.01) |  | 1.17 (-5.66, 8.01) |  |
| PFNA | Internalizing Problems | 1 | 0.00 (reference) | 0.36 | 0.00 (reference) | 0.36 | 0.00 (reference) | 0.82 | 0.00 (reference) | 0.82 |
|  |  | 2 | -1.02 (-2.02, 0.09) |  | -1.02 (-2.02, 0.10) |  | 0.59 (-0.54, 1.71) |  | 0.59 (-0.54, 1.72) |  |
|  |  | 3 | -0.78 (-1.85, 0.38) |  | -0.78 (-1.86, 0.38) |  | -0.02 (-1.47, 1.42) |  | -0.02 (-1.46, 1.43) |  |
|  |  | 4 | -0.84 (-2.11, 0.45) |  | -0.84 (-2.12, 0.46) |  | -0.14 (-1.64, 1.35) |  | -0.14 (-1.64, 1.36) |  |
|  | Externalizing Problems | 1 | 0.00 (reference) | 0.22 | 0.00 (reference) | 0.20 | 0.00 (reference) | 0.98 | 0.00 (reference) | 0.99 |
|  |  | 2 | -0.61 (-1.88, 0.67) |  | -0.62 (-1.90, 0.65) |  | 0.22 (-1.15, 1.58) |  | 0.26 (-1.11, 1.63) |  |
|  |  | 3 | -0.72 (-2.08, 0.65) |  | -0.74 (-2.11, 0.62) |  | 0.16 (-1.59, 1.91) |  | 0.18 (-1.56, 1.93) |  |
|  |  | 4 | -1.07 (-2.69, 0.56) |  | -1.09 (-2.71, 0.53) |  | -0.08 (-1.89, 1.73) |  | -0.06 (-1.87, 1.76) |  |
|  | Total Problems | 1 | 0.00 (reference) | 0.22 | 0.00 (reference) | 0.21 | 0.00 (reference) | 0.94 | 0.00 (reference) | 0.93 |
|  |  | 2 | -2.32 (-5.51, 0.89) |  | -2.35 (-5.53, 0.93) |  | 1.99 (-2.05, 6.03) |  | 2.05 (-2.00, 6.10) |  |
|  |  | 3 | -2.07 (-5.49, 1.35) |  | -2.13 (-5.54, 1.31) |  | 0.11 (-5.07, 5.29) |  | 0.14 (-5.04, 5.32) |  |
|  |  | 4 | -2.94 (-7.00, 1.12) |  | -2.99 (-7.05, 1.07) |  | 0.39 (-4.97, 5.76) |  | 0.43 (-4.94, 5.79) |  |
| PFOS | Internalizing Problems | 1 | 0.00 (reference) | 0.46 | 0.00 (reference) | 0.45 | 0.00 (reference) | 0.80 | 0.00 (reference) | 0.80 |
|  |  | 2 | -0.32 (-1.25, 0.60) |  | -0.32 (-1.25, 0.60) |  | -0.27 (-1.34, 0.79) |  | -0.28 (-1.35, 0.80) |  |
|  |  | 3 | 0.26 (-0.70, 1.23) |  | 0.26 (-0.70, 1.23) |  | 0.25 (-1.14, 1.63) |  | 0.25 (-1.14, 1.63) |  |
|  |  | 4 | 0.28 (-0.97, 1.53) |  | 0.28 (-0.96, 1.53) |  | -0.7 (-2.79, 1.39) |  | -0.70 (-2.80, 1.40) |  |
|  | Externalizing Problems | 1 | 0.00 (reference) | 0.62 | 0.00 (reference) | 0.62 | 0.00 (reference) | 0.73 | 0.00 (reference) | 0.67 |
|  |  | 2 | -0.33 (-1.50, 0.84) |  | -0.34 (-1.51, 0.83) |  | -0.44 (-1.73, 0.84) |  | -0.40 (-1.69, 0.89) |  |
|  |  | 3 | -0.20 (-1.42, 1.02) |  | -0.22 (-1.44, 0.99) |  | 0.53 (-1.14, 2.20) |  | 0.57 (-1.11, 2.24) |  |
|  |  | 4 | -0.48 (-2.06, 1.10) |  | -0.45 (-2.03, 1.13) |  | 0.16 (-2.36, 2.68) |  | 0.24 (-2.29, 2.77) |  |
|  | Total Problems | 1 | 0.00 (reference) | 0.92 | 0.00 (reference) | 0.92 | 0.00 (reference) | 0.85 | 0.00 (reference) | 0.83 |
|  |  | 2 | -0.91 (-3.84, 2.03) |  | -0.93 (-3.86, 2.00) |  | -0.97 (-4.79, 2.86) |  | -0.93 (-4.76, 2.91) |  |
|  |  | 3 | 0.31 (-2.74, 3.36) |  | 0.26 (-2.78, 3.31) |  | 1.59 (-3.37, 6.54) |  | 1.62 (-3.34, 6.59) |  |
|  |  | 4 | -0.92 (-4.87, 3.04) |  | -0.86 (-4.81, 3.09) |  | -0.77 (-8.26, 6.71) |  | -0.70 (-8.21, 6.80) |  |
| PFOA | Internalizing Problems | 1 | 0.00 (reference) | 0.27 | 0.00 (reference) | 0.26 | - |  | - |  |
|  |  | 2 | -0.82 (-1.90, 0.23) |  | -0.82 (-0.82, -0.82) |  |  |  |  |  |
|  |  | 3 | -0.47 (-1.61, 0.66) |  | -0.47 (-0.47, -0.47) |  |  |  |  |  |
|  |  | 4 | -0.95 (-2.20, 0.31) |  | -0.96 (-0.96, -0.96) |  |  |  |  |  |
|  | Externalizing Problems | 1 | 0.00 (reference) | 0.16 | 0.00 (reference) | 0.14 | - |  | - |  |
|  |  | 2 | -0.26 (-1.55, 1.03) |  | -0.31 (-0.31, -0.31) |  |  |  |  |  |
|  |  | 3 | -0.37 (-1.77, 1.04) |  | -0.40 (-0.40, -0.40) |  |  |  |  |  |
|  |  | 4 | -1.18 (-2.77, 0.41) |  | -1.25 (-1.25, -1.25) |  |  |  |  |  |
|  | Total Problems | 1 | 0.00 (reference) | 0.20 | 0.00 (reference) | 0.18 | - |  | - |  |
|  |  | 2 | -1.99 (-5.22, 1.24) |  | -2.08 (-2.08, -2.08) |  |  |  |  |  |
|  |  | 3 | -1.27 (-4.78, 2.24) |  | -1.33 (-1.33, -1.33) |  |  |  |  |  |
|  |  | 4 | -3.13 (-7.11, 0.84) |  | -3.28 (-3.28, -3.28) |  |  |  |  |  |

^a^All models were adjusted for child sex, child age at CBCL assessment, birth year, maternal age at delivery, maternal race and ethnicity, maternal pre-pregnancy BMI, maternal educational attainment, parity, marital status, and cohort site. These models were fit among the subset of participants with data on maternal smoking during pregnancy.

BMI, body mass index; CBCL, Child Behavior Checklist; PFAS, per- and polyfluoroalkyl substances; PFHxS, perfluorohexane sulfonic acid; PFNA, perfluorononanoic acid; PFOA, perfluorooctanoic acid; PFOS, perfluorooctane sulfonic acid.

| **Table S9.** Interaction p-values for sex differences in adjusted associations between PFAS detected at >75% frequency in prenatal maternal serum and raw CBCL scores among preschool-age and school-age children^a,b^ | | | | | | |
| --- | --- | --- | --- | --- | --- | --- |
|  | **Preschool-Age subset**  **(N=1,505)** | | | **School-Age subset**  **(N=581)** | | |
| **PFAS** | **Internalizing Problems** | **Externalizing Problems** | **Total**  **Problems** | **Internalizing Problems** | **Externalizing Problems** | **Total**  **Problems** |
| PFHxS | 0.21 | 0.64 | 0.36 | 0.35 | 0.26 | 0.38 |
| PFOS | 0.84 | 0.97 | 0.87 | 0.24 | 0.21 | 0.35 |
| PFNA | 0.46 | 0.21 | 0.17 | 0.23 | 0.23 | 0.24 |
| PFOA | 0.60 | 0.90 | 0.81 | - | - | - |
| ^a^PFOA was detected in fewer than 75% of maternal prenatal serum samples corresponding to school-age children and thus was not tested for effect modification by sex.  ^b^Models were adjusted for child sex, child age at CBCL assessment, birth year, maternal age at delivery, maternal race and ethnicity, maternal pre-pregnancy BMI, maternal educational attainment, parity, marital status, and cohort site (using random intercepts). | | | | | | |

BMI, body mass index; CBCL, Child Behavior Checklist; PFAS, per- and polyfluoroalkyl substances; PFHxS, perfluorohexane sulfonic acid; PFNA, perfluorononanoic acid; PFOA, perfluorooctanoic acid; PFOS, perfluorooctane sulfonic acid.

**Figure S1.** Directed acyclic graph depicting the hypothesized relationships among prenatal maternal PFAS exposure, childhood behavioral problems, and covariates
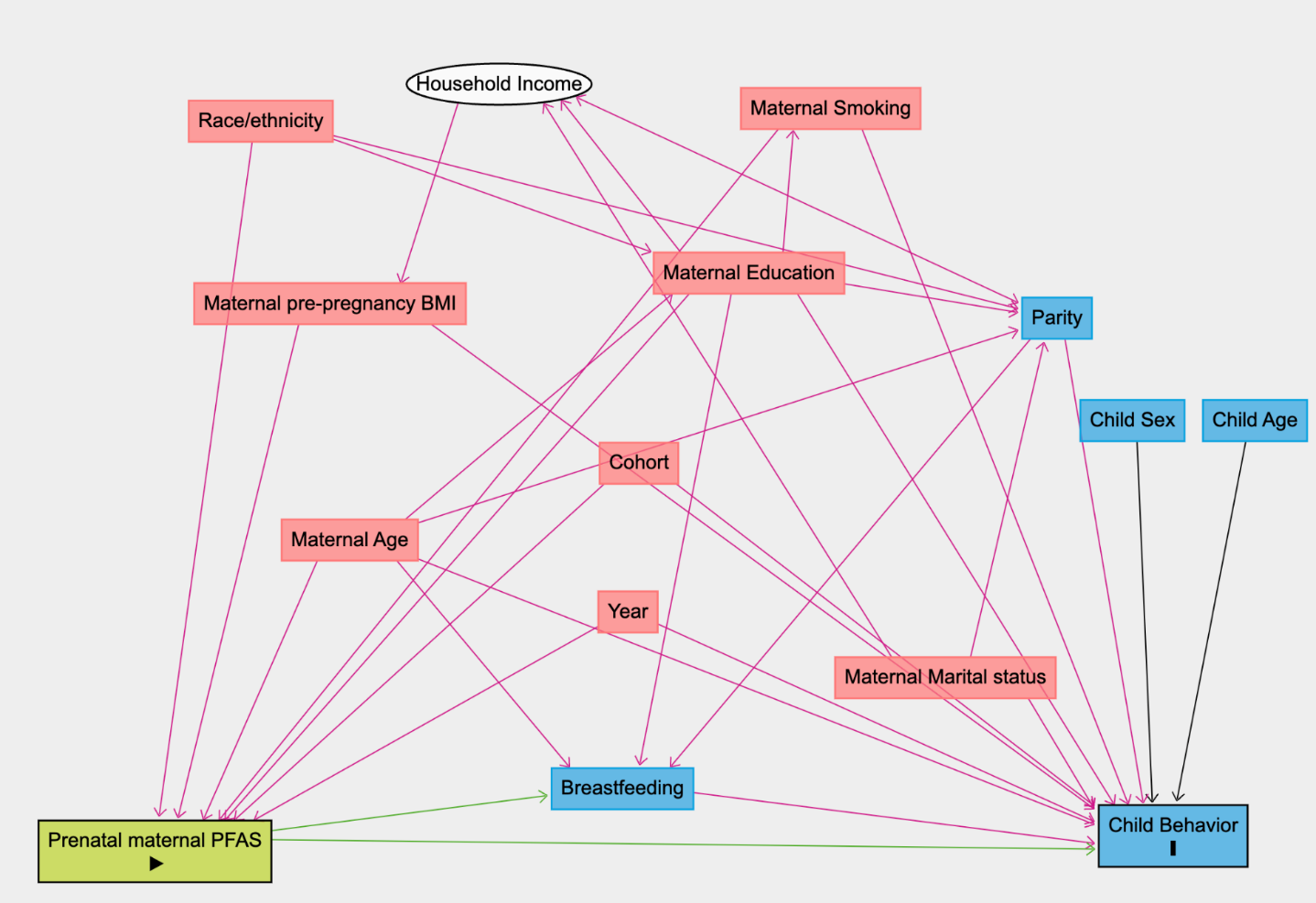


BMI, body mass index; PFAS, per- and polyfluoroalkyl substances. The green box shows the exposure of interest (prenatal maternal PFAS); the blue boxes depict the outcome of interest (child behavior) or ancestors thereof; and the red boxes depict ancestors of both the exposure and outcome (*i.e.,* potential confounders).

**Figure S2.** Flow diagram of inclusion and exclusion criteria used to define the final analytic samples
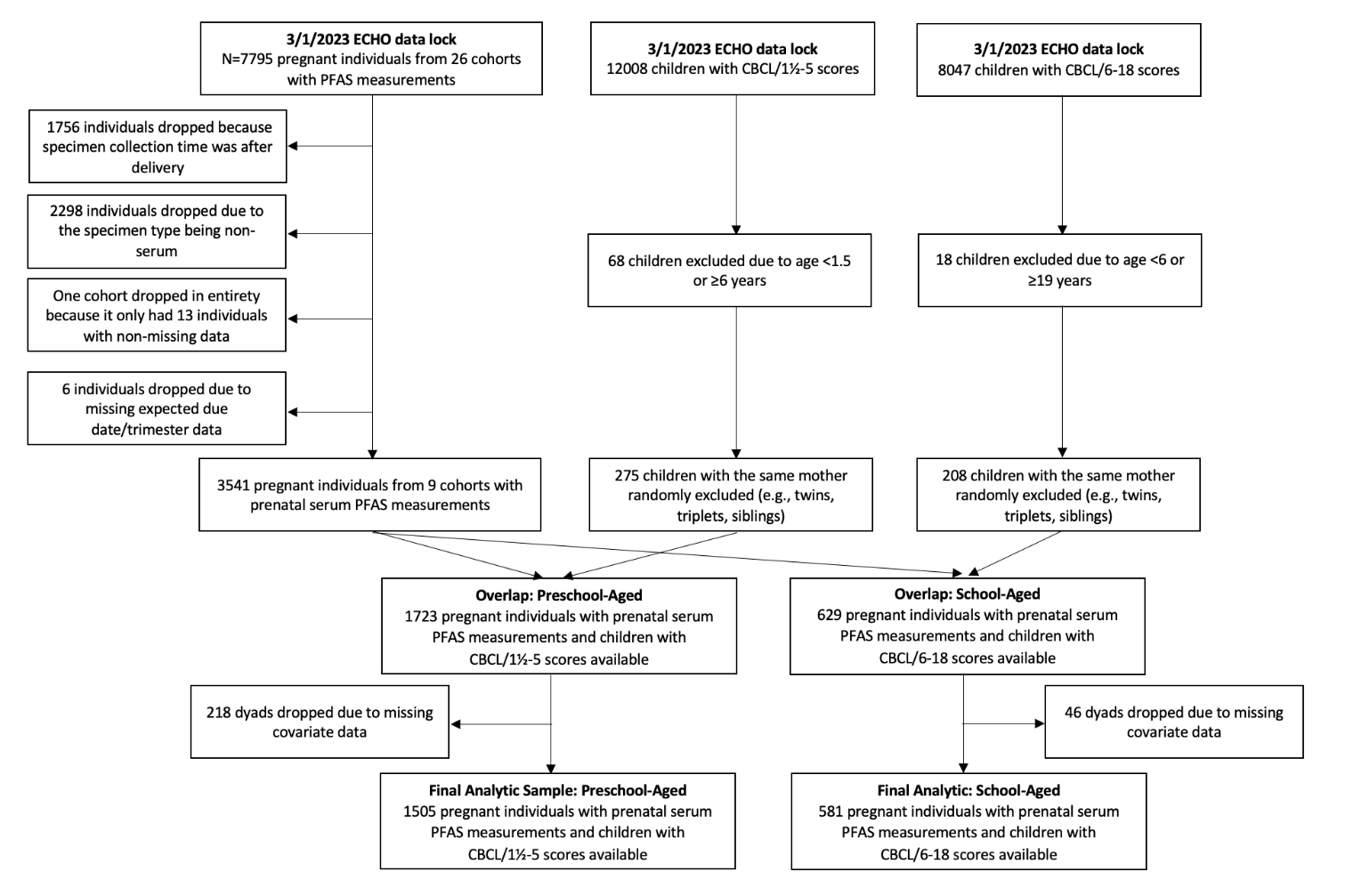


CBCL, Child Behavior Checklist; PFAS, per- and polyfluoroalkyl substances.

**Figure S3.** Associations between PFAS detected at >75% frequency in prenatal maternal serum samples and raw CBCL scores among preschool-age children (1.9-5 years of age) after adjustment for child sex, child age at CBCL assessment, birth year, maternal age at delivery, maternal race and ethnicity, maternal pre-pregnancy BMI, maternal educational attainment, parity, marital status, and cohort site (N=1,505)


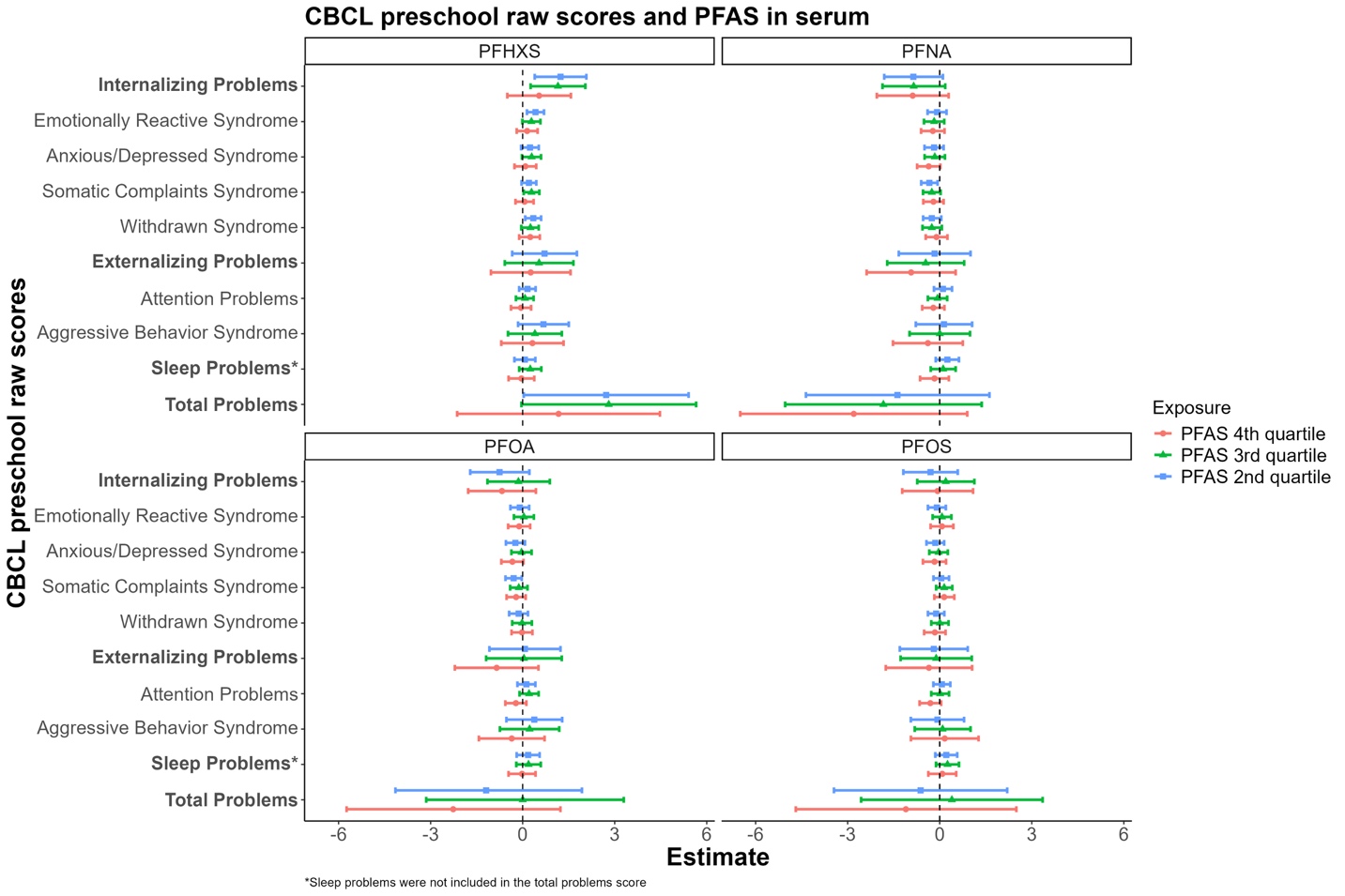


BMI, body mass index; CBCL, Child Behavior Checklist; PFAS, per- and polyfluoroalkyl substances; PFHxS, perfluorohexane sulfonic acid; PFNA, perfluorononanoic acid; PFOA, perfluorooctanoic acid; PFOS, perfluorooctane sulfonic acid.

**Figure S4.** Associations of PFOA and PFNA in second-trimester maternal serum samples with normalized CBCL T-scores among preschool-age children (1.9-5 years of age) after adjustment for child sex, child age at CBCL assessment, birth year, maternal age at delivery, maternal race and ethnicity, maternal pre-pregnancy BMI, maternal educational attainment, parity, marital status, and cohort site (N=953)

**
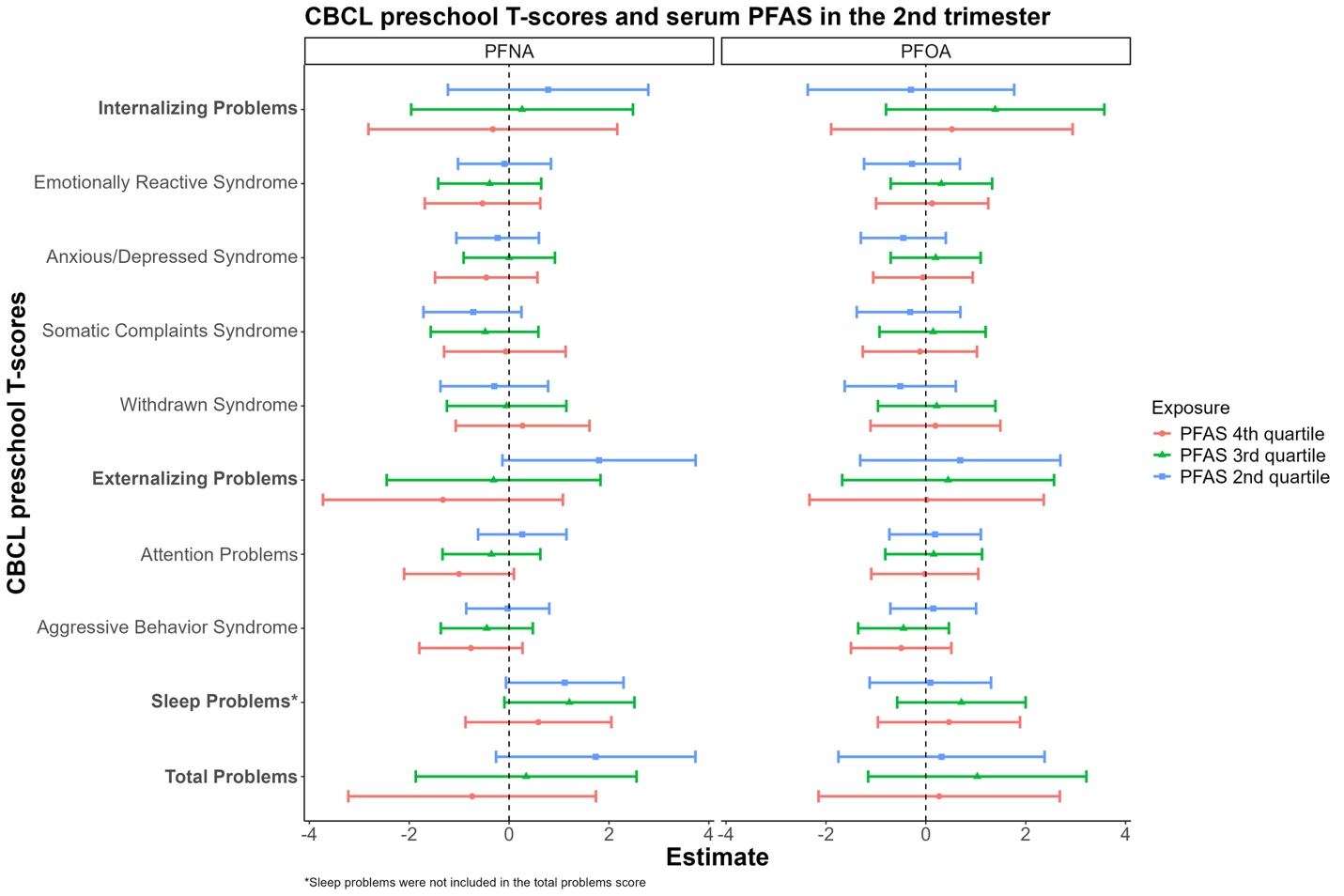
**

BMI, body mass index; CBCL, Child Behavior Checklist; PFNA, perfluorononanoic acid; PFOA, perfluorooctanoic acid.

* denotes a p_trend_ < 0.05.

**Figure S5.** Associations between PFAS detected at <75% frequency in prenatal maternal serum samples and CBCL T-scores among preschool-age children (1.9-5 years of age) after adjustment for child sex, child age at CBCL assessment, birth year, maternal age at delivery, maternal race and ethnicity, maternal pre-pregnancy BMI, maternal educational attainment, parity, marital status, and cohort site (N=1,505)


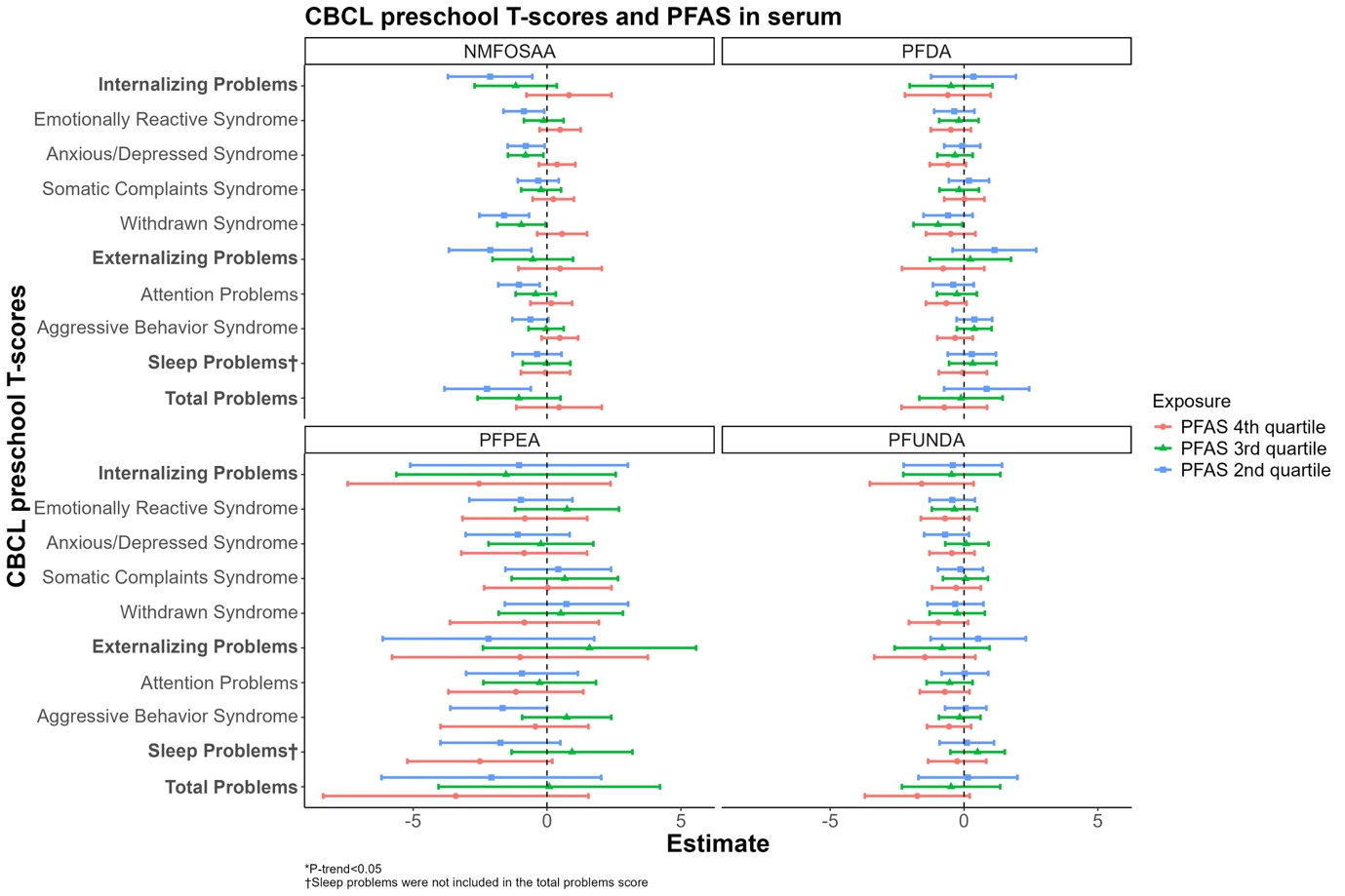


BMI, body mass index; CBCL, Child Behavior Checklist; PFAS, per- and polyfluoroalkyl substances; NMFOSAA/MeFOSAA, N-methyl perfluorooctanesulfonamidoacetic acid; PFDA, perfluorodecanoic acid; PFDS, perfluorodecanesulfonic acid; PFPEA, perfluoropentanoic acid; PFUNDA, perfluoroundecanoic acid.

**Figure S6.** Associations between PFAS detected at <75% frequency in prenatal maternal serum samples and CBCL raw scores among preschool-age children (1.9-5 years of age) after adjustment for child sex, child age at CBCL assessment, birth year, maternal age at delivery, maternal race and ethnicity, maternal pre-pregnancy BMI, maternal educational attainment, parity, marital status, and cohort site (N=1,505)


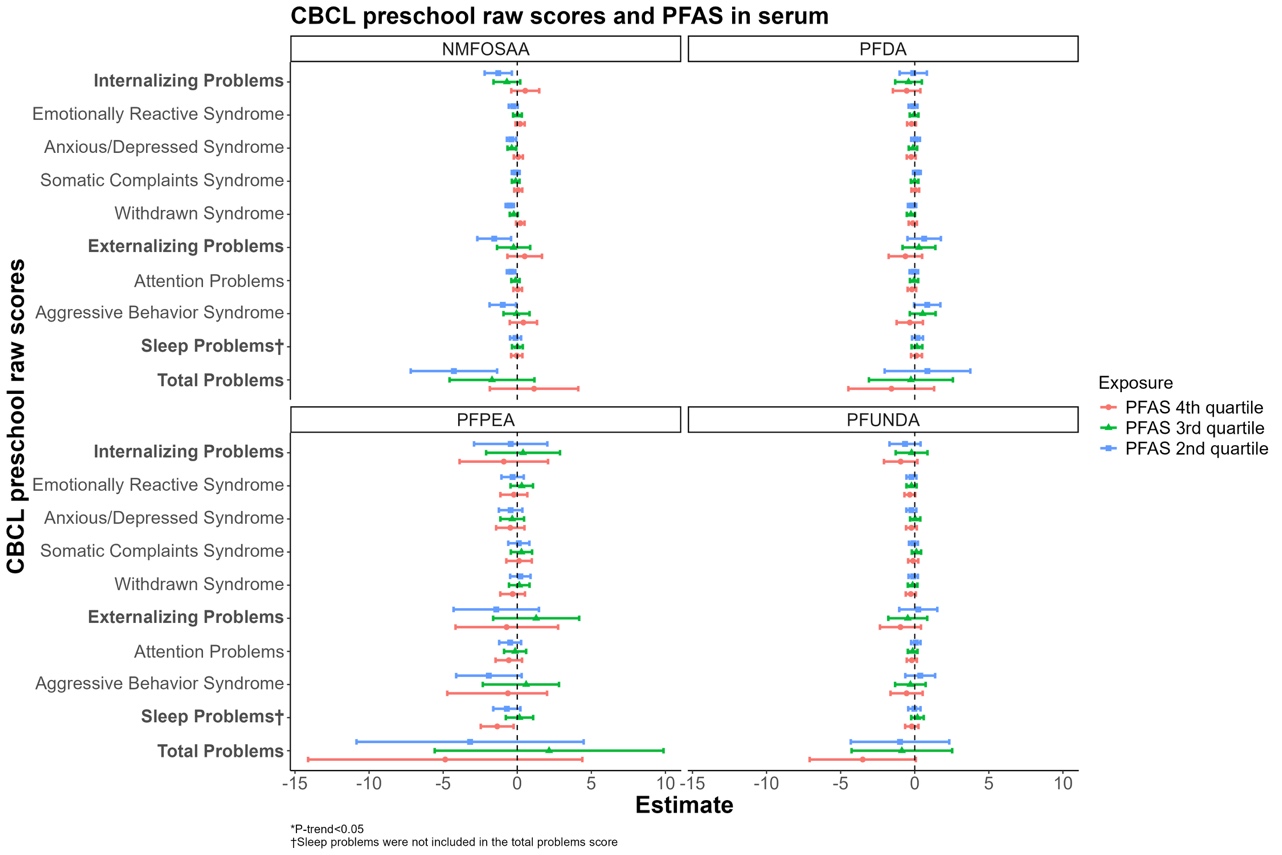


BMI, body mass index; CBCL, Child Behavior Checklist; PFAS, per- and polyfluoroalkyl substances; NMFOSAA/MeFOSAA, N-methyl perfluorooctanesulfonamidoacetic acid; PFDA, perfluorodecanoic acid; PFPEA, perfluoropentanoic acid; PFUNDA, perfluoroundecanoic acid.

**Figure S7.** Associations between PFAS detected at >75% frequency in prenatal maternal serum samples and raw CBCL scores among school-age children (6-15 years of age) after adjustment for child sex, child age at CBCL assessment, birth year, maternal age at delivery, maternal race and ethnicity, maternal pre-pregnancy BMI, maternal educational attainment, parity, marital status, and cohort site (N=581)


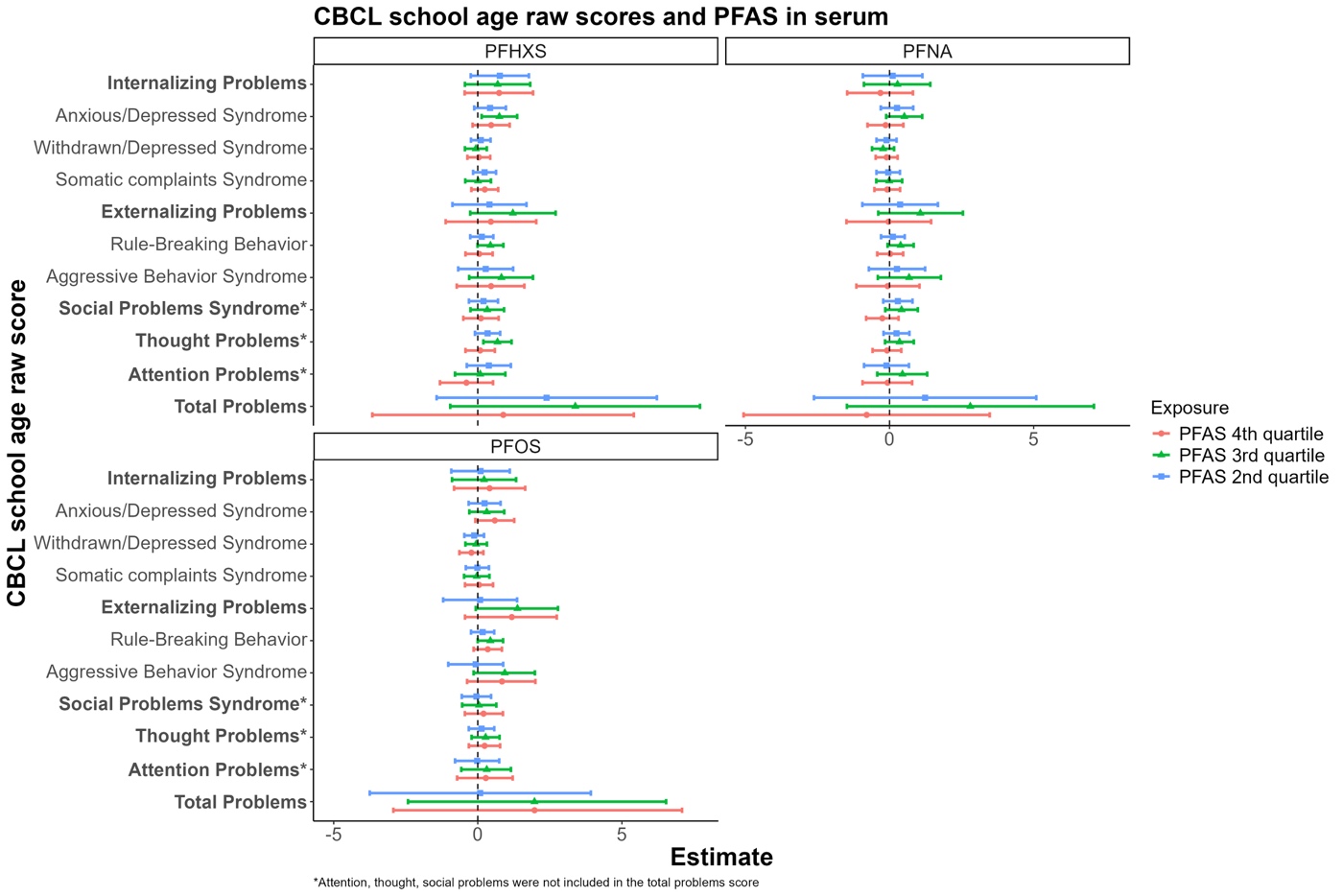


BMI, body mass index; CBCL, Child Behavior Checklist; PFAS, per- and polyfluoroalkyl substances; PFHxS, perfluorohexane sulfonic acid; PFNA, perfluorononanoic acid; PFOS, perfluorooctane sulfonic acid.
* denotes a p_trend_ < 0.05.

**Figure S8.** Associations of PFOA and PFNA in second-trimester maternal serum samples with normalized CBCL T-scores among school-age children (6-15 years of age) after adjustment for child sex, child age at CBCL assessment, birth year, maternal age at delivery, maternal race and ethnicity, maternal pre-pregnancy BMI, maternal educational attainment, parity, marital status, and cohort site (N=391)
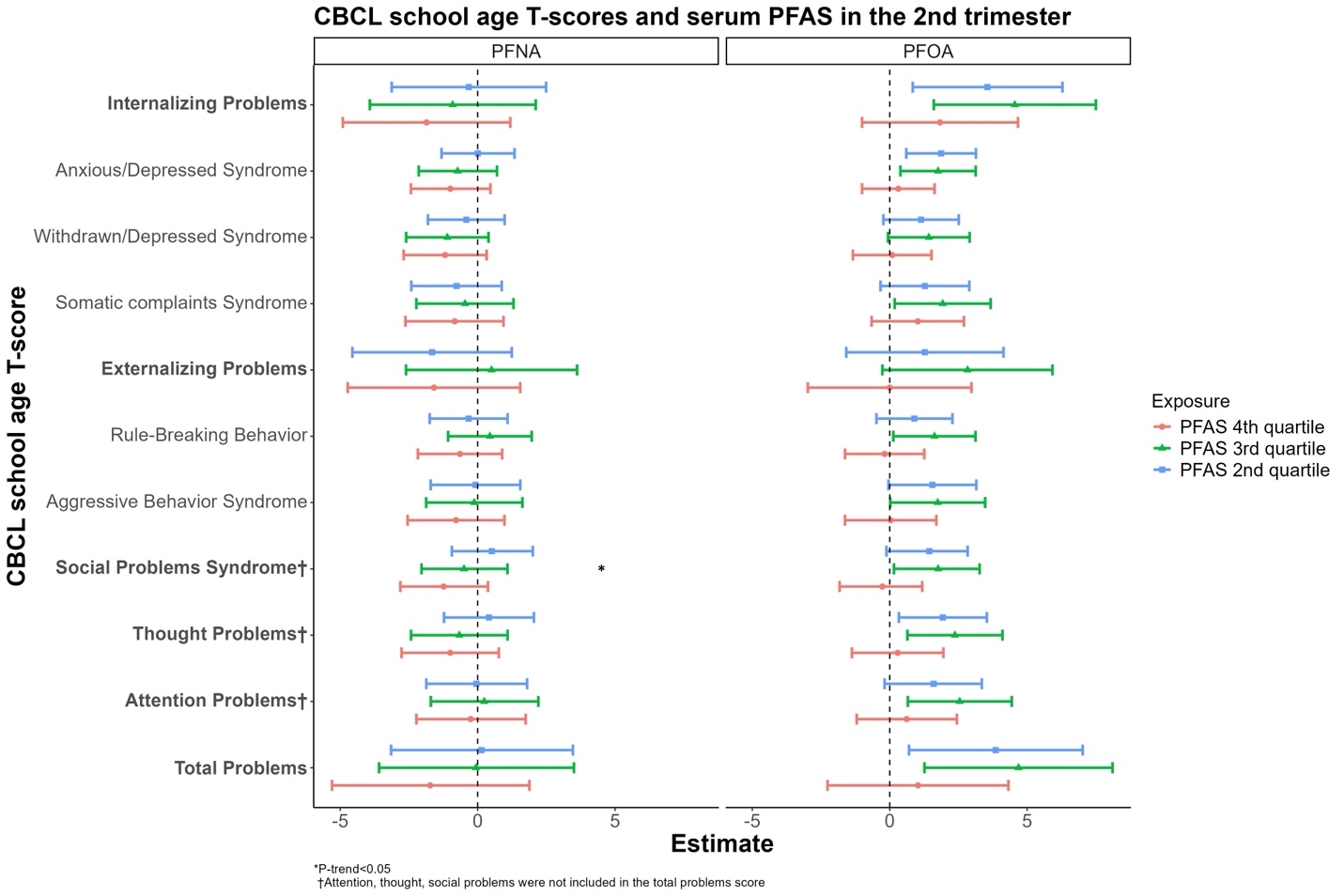


BMI, body mass index; CBCL, Child Behavior Checklist; PFNA, perfluorononanoic acid; PFOA, perfluorooctanoic acid.

* denotes a p_trend_ < 0.05.

**Figure S9.** Associations between PFAS detected at <75% frequency in prenatal maternal serum samples and CBCL T-scores among school-age children (6-15 years of age) after adjustment for child sex, child age at CBCL assessment, birth year, maternal age at delivery, maternal race and ethnicity, maternal pre-pregnancy BMI, maternal educational attainment, parity, marital status, and cohort site (N=581)


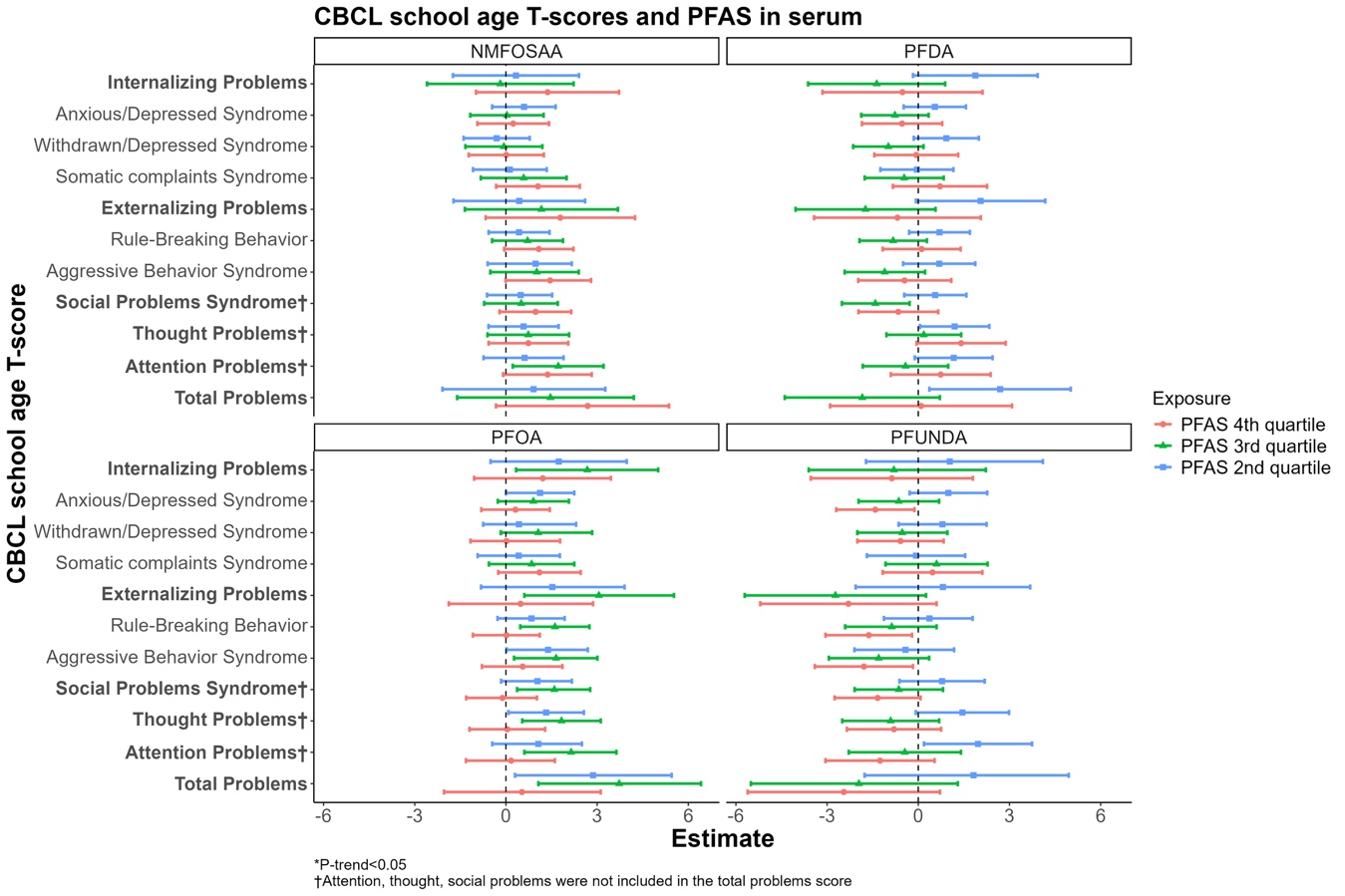


BMI, body mass index; CBCL, Child Behavior Checklist; PFAS, per- and polyfluoroalkyl substances; NMFOSAA/MeFOSAA, N-methyl perfluorooctanesulfonamidoacetic acid; PFDA, perfluorodecanoic acid; PFDS, perfluorodecanesulfonic acid; PFOA, perfluorooctanoic acid; PFUNDA, perfluoroundecanoic acid.
* denotes a p_trend_ < 0.05.

**Figure S10.** Associations between PFAS detected at <75% frequency in prenatal maternal serum samples and CBCL raw scores among school-age children (6-15 years of age) after adjustment for child sex, child age at CBCL assessment, birth year, maternal age at delivery, maternal race and ethnicity, maternal pre-pregnancy BMI, maternal educational attainment, parity, marital status, and cohort site (N=581)


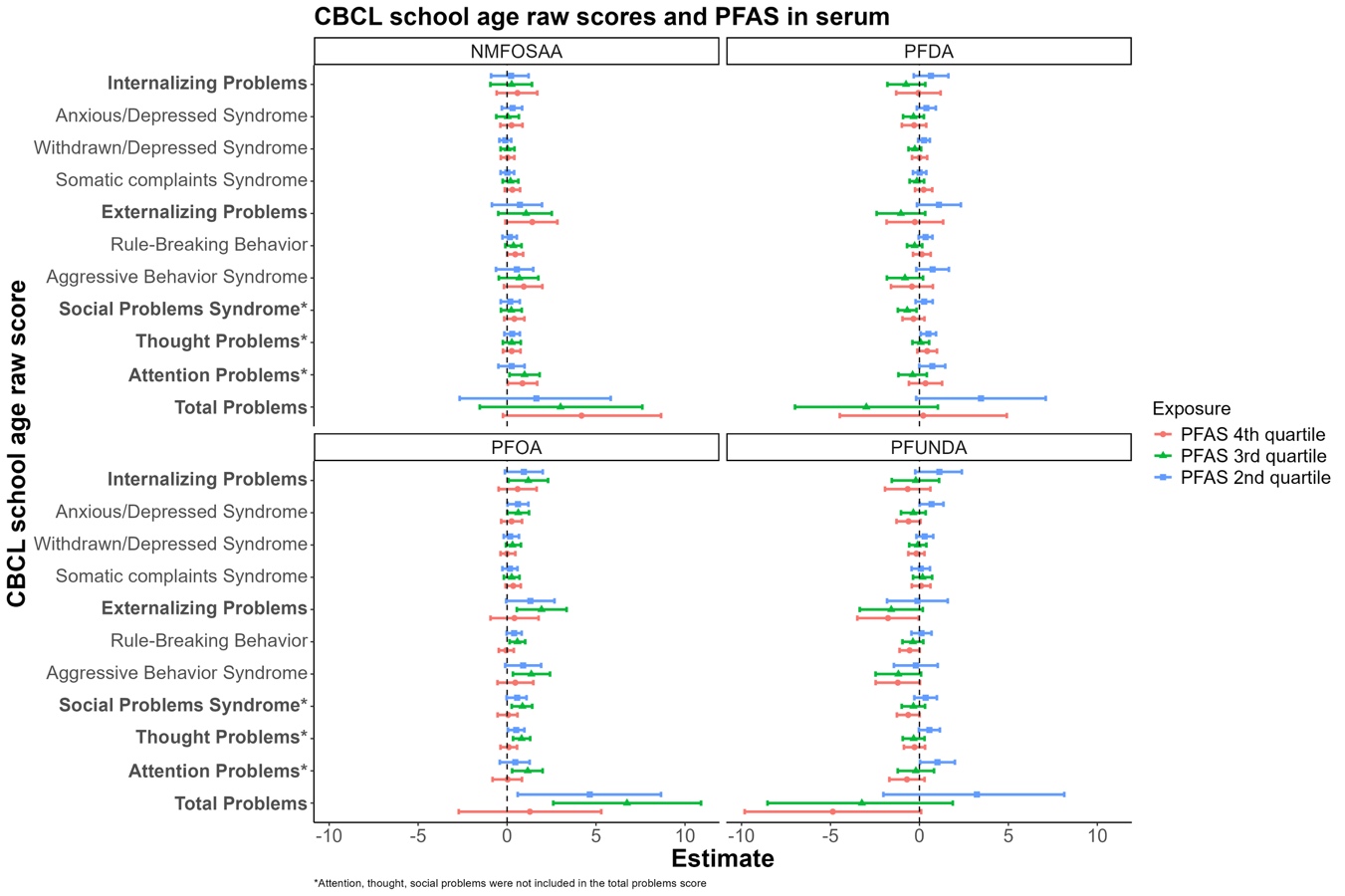


BMI, body mass index; CBCL, Child Behavior Checklist; PFAS, per- and polyfluoroalkyl substances; NMFOSAA/MeFOSAA, N-methyl perfluorooctanesulfonamidoacetic acid; PFDA, perfluorodecanoic acid; PFDS, perfluorodecanesulfonic acid; PFOA, perfluorooctanoic acid; PFUNDA, perfluoroundecanoic acid.

**Figure S11.** Leave-one-out analysis for prenatal PFHxS quartiles and select CBCL T-scores among the preschool-age subset across the nine ECHO Cohort study sites


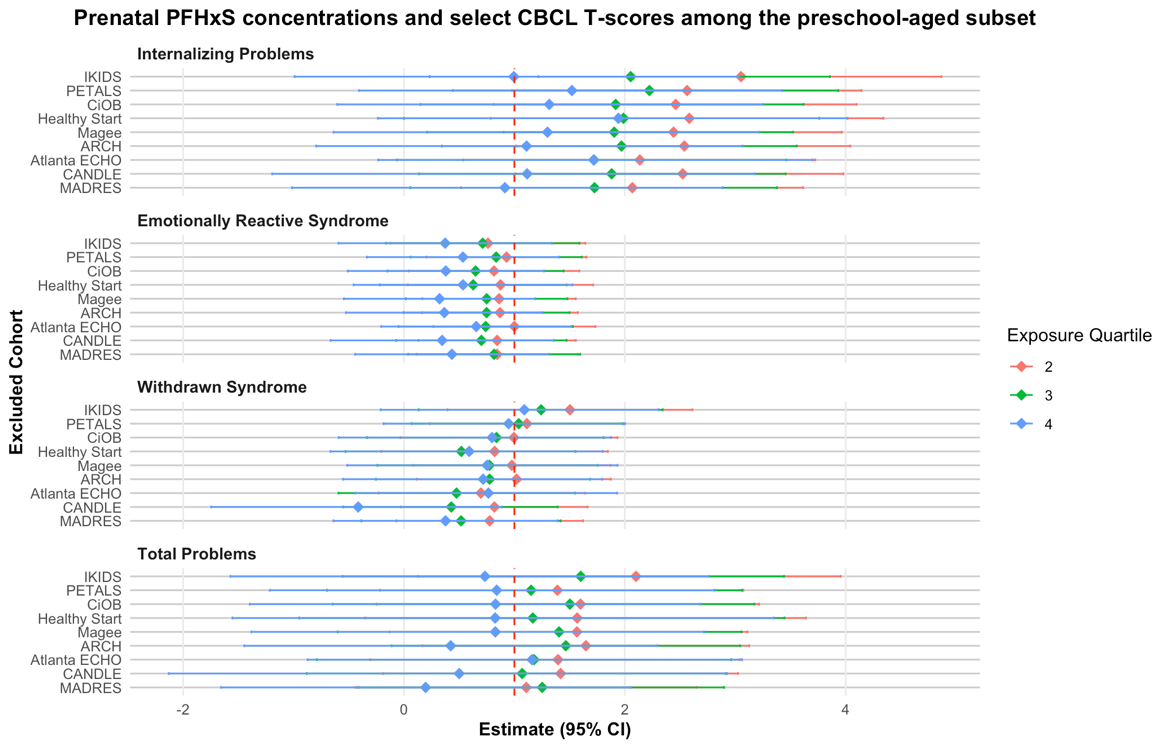


CBCL, Child Behavior Checklist; PFHxS, perfluorohexane sulfonic acid.

**Figure S12.** Leave-one-out analysis for prenatal PFOS quartiles and externalizing problems CBCL T-scores among the school-age subset across the six ECHO Cohort study sites


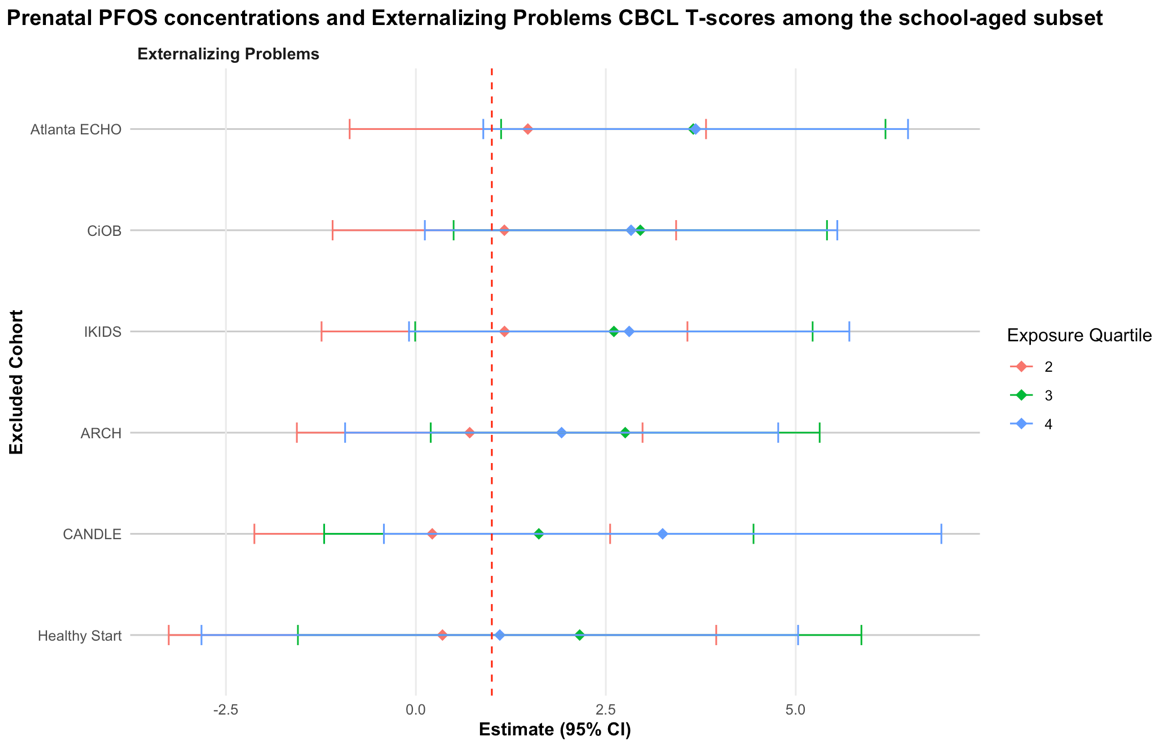


CBCL, Child Behavior Checklist; PFOS, perfluorooctane sulfonic acid.

**Figure S13.** Leave-one-out analysis for prenatal PFOA quartiles and select CBCL T-scores among the school-age subset across the six ECHO Cohort study sites


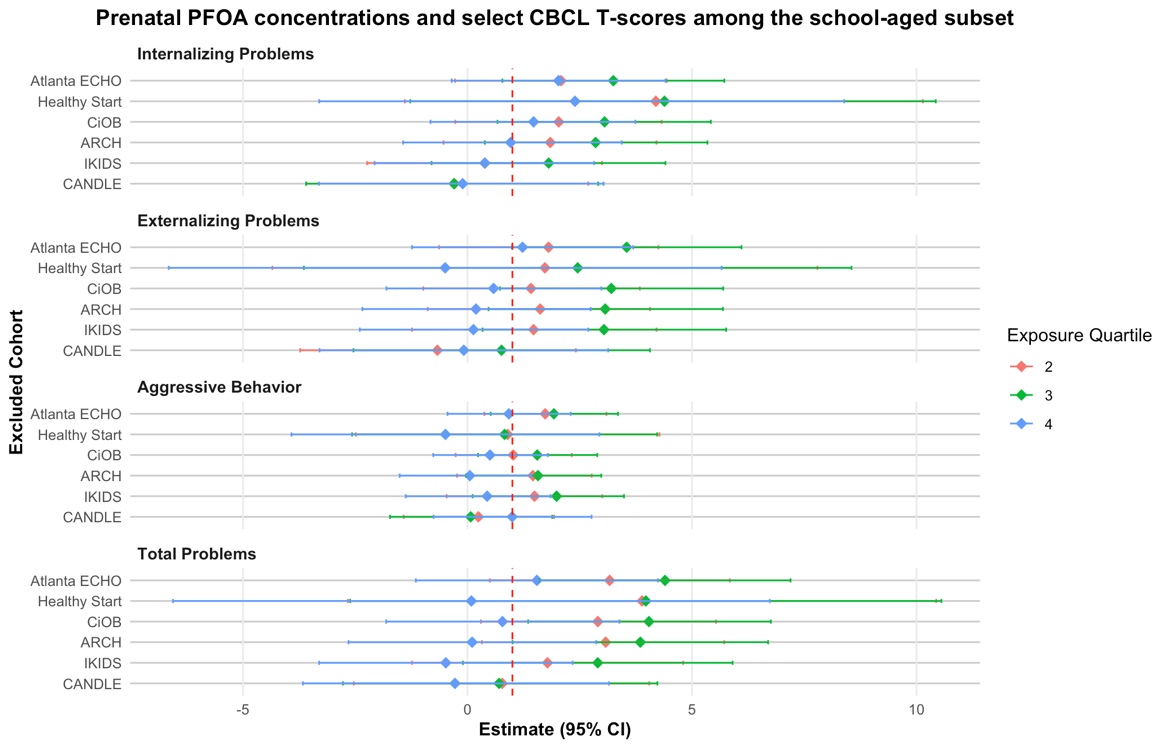


CBCL, Child Behavior Checklist; PFOA, perfluorooctanoic acid.
